# Have e-cigarettes renormalised or displaced youth smoking? Results of a segmented regression analysis of repeated cross sectional survey data in England, Scotland and Wales

**DOI:** 10.1136/tobaccocontrol-2018-054584

**Published:** 2019-04-01

**Authors:** Britt Hallingberg, Olivia M Maynard, Linda Bauld, Rachel Brown, Linsay Gray, Emily Lowthian, Anne-Marie MacKintosh, Laurence Moore, Marcus R Munafo, Graham Moore

**Affiliations:** 1 Centre for the Development and Evaluation of Complex Interventions for Public Health Improvement, School of Social Sciences, Cardiff University, Cardiff, UK; 2 School of Experimental Psychology, University of Bristol, Bristol, UK; 3 Usher Institute and UK Centre for Tobacco and Alcohol Studies, College of Medicine and Veterinary Medicine, University of Edinburgh, Edinburgh, UK; 4 MRC/CSO Social & Public Health Sciences Unit, University of Glasgow, Glasgow, UK; 5 Centre for Tobacco Control Research, Institute for Social Marketing, University of Stirling, Stirling, UK

**Keywords:** electronic nicotine delivery devices, harm reduction, public policy, denormalization

## Abstract

**Objectives:**

To examine whether during a period of limited e-cigarette regulation and rapid growth in their use, smoking began to become renormalised among young people.

**Design:**

Interrupted time-series analysis of repeated cross-sectional time-series data.

**Setting:**

Great Britain

**Participants:**

248 324 young people aged approximately 13 and 15 years, from three national surveys during the years 1998–2015.

**Intervention:**

Unregulated growth of e-cigarette use (following the year 2010, until 2015).

**Outcome measures:**

Primary outcomes were prevalence of self-reported ever smoking and regular smoking. Secondary outcomes were attitudes towards smoking. Tertiary outcomes were ever use of cannabis and alcohol.

**Results:**

In final models, no significant change was detected in the pre-existing trend for ever smoking (OR 1.01, CI 0.99 to 1.03). There was a marginally significant slowing in the rate of decline for regular smoking (OR 1.04, CI 1.00 to 1.08), accompanied by a larger slowing in the rate of decline of cannabis use (OR 1.21, CI 1.18 to 1.25) and alcohol use (OR 1.17, CI 1.14 to 1.19). In all models and subgroup analyses for smoking attitudes, an increased rate of decline was observed after 2010 (OR 0.88, CI 0.86 to 0.90). Models were robust to sensitivity analyses.

**Conclusions:**

There was a marginal slowing in the decline in regular smoking during the period following 2010, when e-cigarettes were emerging but relatively unregulated. However, these patterns were not unique to tobacco use and the decline in the acceptability of smoking behaviour among youth accelerated during this time. These analyses provide little evidence that renormalisation of youth smoking was occurring during a period of rapid growth and limited regulation of e-cigarettes from 2011 to 2015.

**Trial registration number:**

Research registry number: researchregistry4336

## Background

Electronic cigarettes, first developed in China, have proliferated in many countries in the last decade. In the UK, adult use of e-cigarettes rose rapidly from 2011 before plateauing from 2013.^1^ Some argue that e-cigarettes appear to have had small, but important, positive population level impacts on adult smoking cessation rates.^2 3^ Although this remains contested,^4 5^ their harm reduction potential has led many to support their use as an alternative to smoking.^6^ However, public health communities remain divided in approaches to harm reduction and views on the extent to which e-cigarettes should be regulated.^7^ While Public Health England has supported less restrictive policies,^8^ the Centre for Disease Control and Prevention (CDC) in the USA has highlighted potential harms of e-cigarettes, supporting a more restrictive approach to their use.^9^ In North America, policies have included banning e-cigarette use wherever tobacco use is prohibited,^7^ while in other countries, such as Australia, sales of e-cigarettes containing nicotine remain illegal,^10^ citing concerns of smoking renormalisation.^11^

Growth of e-cigarette use among young people has been framed to some extent as a potential public health problem in its own right, due to some evidence from animal models that nicotine may impair adolescent brain development.^12^ However, the most commonly expressed concern among those calling for greater regulation relates to their potential impact on young people’s smoking. Unlike adult use of e-cigarettes which has largely been limited to smokers or ex-smokers,^13^ emerging international evidence indicates increasing numbers of adolescents who have never used tobacco are experimenting with e-cigarettes.^14–16^ These studies show that by 2015, experimentation with e-cigarettes was more common than experimentation with tobacco. Notably, they also show that experimentation is not translating into widespread regular e-cigarette use to date.^17 18^ Nevertheless, a perception that e-cigarette proliferation may renormalise smoking,^19^ through leading young people to view smoking as a socially acceptable behaviour, has been cited in policy documents in several countries as a rationale to support more restrictive policies. The European Union (EU) Tobacco Products Directive (TPD)^20^ has regulated e-cigarettes in partial alignment with tobacco, contending “Electronic cigarettes can develop into a gateway to nicotine addiction and ultimately traditional tobacco consumption, as they mimic and normalise the action of smoking. For this reason, it is appropriate to adopt a restrictive approach to advertising electronic cigarettes and refill containers” (page 7^20^). The Australian government has stated: “…the Department is concerned about evidence suggesting that e-cigarettes may provide a gateway to nicotine addiction or tobacco use (particularly among youth), and may re-normalise smoking” (page 1^21^).

Much success in maintaining a continuous downward trajectory in youth smoking in the past 20 years has been achieved through policies that aim to reverse the normalisation of smoking.^22^ The renormalisation hypothesis^23^ assumes that growing prevalence and visibility of e-cigarette use will reverse tobacco control successes through increasing the extent to which smoking is once again seen as a ‘normal’ behaviour, accepted and accommodated by the non-smoking majority, including young people. However, the hypothesis that e-cigarettes will renormalise smoking in young people is premised on an assumption that tobacco use and e-cigarette use are viewed by young people as sufficiently similar for one to renormalise the other. By contrast, some argue that e-cigarettes may denormalise smoking,^24^ through social display of an alternative behaviour, leading to displacement away from tobacco use for some young people who would otherwise have become smokers. From this perspective, alignment of e-cigarettes with tobacco in terms of regulatory frameworks paradoxically risks creating a perception that they are synonymous, potentially creating conditions for renormalisation to occur.

To date, national surveys in a number of countries have shown that smoking rates among young people have continued to fall in recent years, despite the growth of e-cigarette use.^17 25–28^ However, few attempts have been made to model whether this decline has occurred at a faster rate (as would be expected were displacement to be taking place), or a slowed rate since the emergence of e-cigarettes (as would be expected were renormalisation to be taking place), or to examine changes in young people’s attitudes toward smoking as a normative behaviour. To date, only one US study has tested these changes in trend, finding no evidence of change in trend for youth smoking during the period of rapid growth, but limited regulation, of e-cigarette use.^29^ The aim of the current study was therefore to examine these competing hypotheses by examining trends of smoking and smoking attitudes of young people in the UK since 1998, with a focus on whether these trends changed significantly after 2010 until 2015—the period of time when e-cigarettes were emerging, but largely unregulated (ie, before the introduction of the EU TPD).^20^ Changes in trend for tobacco use and smoking attitudes were accompanied by analyses of trends for alcohol and cannabis use, to examine the extent to which change in trend during this period is unique to tobacco or reflective of broader substance use trajectories which are less likely to have changed as a direct consequence of e-cigarettes.

## Methods

### Population-sampled data

Nationally representative samples of secondary school students were used from England, Scotland and Wales from the following repeated cross-sectional surveys: the annual Smoking Drinking and Drug Use Among Young People in England Survey (SDDU), the biennial Scottish Adolescent Lifestyle and Substance Use Survey (SALSUS), and for Wales, the Health Behaviour in School-aged Children (HBSC) survey (from 1998 to 2013) and the School Health Research Network (SHRN) survey (2015). The HBSC survey takes place every 2 to 4 years, with the SHRN survey developed from the 2013 survey and an SHRN survey conducted in 2015 (as of 2017, HBSC is integrated into the larger SHRN survey). Further details about sampling strategies and procedures used for these surveys, including access to SDDU and SALSUS data, are available elsewhere.^26 27 30–48^

### Outcome measures

#### Sociodemographic information

All surveys asked young people to indicate whether they were male or female. SALSUS only surveys pupils in S2 and S4 (ie, pupils aged approximately 13 and 15 years, respectively). SDDU and HBSC/SHRN datasets collect data from 11 to 16 year olds, but for comparability with Scotland were limited to approximately equivalent school year groups (ie, years 9 and 11). As not all surveys provide an age variable, year group was used as a proxy for age. In SALSUS and SDDU, socioeconomic status (SES) was indicated by a binary variable representing whether or not students reported receiving free school meals. In HBSC/SHRN, the Family Affluence Scale (FAS),^49^ which measures material affluence, was used to indicate SES. As material markers of deprivation shift substantially over time, a relative measure of SES was derived whereby the sample was divided into ‘high’ and ‘low’ affluence within the survey year in question.

#### Primary outcomes: ever smoking and regular smoking

Two binary variables were derived to indicate whether students had ever smoked and whether they smoked regularly (ie, weekly or more). In SDDU and SALSUS, participants were asked to indicate which of the following statements best described them: ‘I have never smoked’, ‘I have only ever smoked once’, ‘I used to smoke but I never smoke a cigarette now’, ‘I sometimes smoke a cigarette now, but I don’t smoke as many as one a week’, ‘I usually smoke between one and six cigarettes a week’, ‘I usually smoke more than six cigarettes a week’. To indicate ever smoking, those who reported ‘I have never smoked’ were compared with all others. For regular use, those who reported smoking between one and six cigarettes a week, or more, were compared with all others. In HBSC/SHRN, students were asked at what age they ‘smoked a cigarette (more than just a puff)’ with the following response options: ‘never’ or a range of ages. Those who reported ‘never’ were compared with all others. Students in HBSC/SHRN were also asked, ‘how often do you smoke at present?’ with response options: ‘every day’, ‘at least once a week but not every day’, ‘less than once a week’ or ‘I do not smoke’. To indicate regular use, those who reported use at least once a week or more frequently were compared with all others.

#### Secondary outcomes: smoking attitudes

In SALSUS (from 2006) and SDDU (from 2003), students were asked ‘Do you think it is OK for someone your age to do the following?: Try a cigarette to see what it is like’. In SDDU in 1999 and 2001, the question wording was slightly different (‘Try smoking once’). Hence, analyses were run with and without these earlier years as a sensitivity analysis. In SDDU only (from 2003), students were also asked whether it was OK for someone their age to smoke cigarettes once a week. Response options for both items were: ‘it’s OK’, ‘it’s not OK’ and ‘I don’t know’. For each smoking attitudes measure, two dichotomous variables were created which coded ‘I don’t know’ as ‘yes’ as well as ‘no’, respectively.

#### Tertiary outcomes: alcohol and cannabis use

Falsifiability checks included replicating analyses for binary indicators of ever alcohol use and ever cannabis use. For alcohol use, both SALSUS and SDDU asked students ‘Have you ever had a proper alcoholic drink – a whole drink, not just a sip?’ with responses of ‘yes’ or ‘no’. From 2002, HBSC/SHRN surveys asked students ‘At what age did you do the following things? If there is something that you have not done, choose the ‘never’ category.’ Responses other than ‘never’ for the category ‘drink alcohol (more than a small amount)’ were classed as ever drinkers. To measure ever cannabis use in SALSUS and SDDU, students were presented with a grid listing a range of drugs and asked which, if any, they have ever used with response options ‘yes’ or ‘no’. In HBSC, pupils were asked how many times they have used cannabis in their lifetime with response options of ‘never’, ‘once to twice’, ‘3 to 5 times’, ‘6 to 9 times’, ‘10 to 19 times’, ‘20 to 39 times’ and ‘40+ times’. A binary variable distinguished ever users from never users.

### Statistical analyses

Segmented time series regression analyses were used. 1998 was selected a priori as the starting time point when youth smoking peaked before commencing a period of approximately linear decline.^50^ Proliferation of e-cigarettes was viewed as a naturally occurring intervention, with 2010 treated as the ‘intervention’ point as surveys of the general population in the UK began to identify emergence of e-cigarette use from 2011.^1^ While not an intervention in the traditional sense of the term, the emergence of e-cigarettes represents an important industry-driven ‘event’ within the tobacco control system with potential to alter its trajectories, positively and negatively.^51^ The following core statistical model was used for *Y_ki_*, the smoking status of individual *i* at time:

$$\begin{array}{ll} k:log[\pi_{ki}/(1-\pi_{ki})]= & \beta 0+\beta 1(time{)}_{ki}+\beta 2(intervention{)}_{ki}+\\ & \beta 3(postslope{)}_{ki}+\beta^{\prime }4(country{)}_{ki}+\varepsilon_{ki} \end{array}$$

where *π_ki_* was the expected value of *Y_ki_*; *time* was a continuous variable indicating time from the start of the study to the end of the period of observation; *intervention* was coded 0 for pre-intervention time points (before, and including, year 2010) and 1 for post-intervention time points (from 2011); and *postslope* was coded 0 up to the last point before the intervention phase and coded sequentially from 1, 2… thereafter. *β_0_* estimated the baseline level of the outcome at time 0 (beginning of the period); *β_1_* estimated the structural trend, independently from the policy intervention; *β_2_* estimated the immediate impact of the intervention and *β_3_* reflects the change in trend/slope after the intervention; *β_4_* is the set of parameters corresponding to the country dummy variables. Data were analysed with all countries’ data combined with year group and gender included as covariates. A *time^2^* covariate was also included to allow for non-linear trajectories in a separate quadratic model. Models were repeated for all outcomes.

Females (as opposed to males), older adolescents and less affluent groups have typically reported higher prevalence rates of smoking, and the role of e-cigarettes in exacerbating or reducing these inequalities is of significant interest. For subgroup analyses, models were therefore stratified by gender, year group, and (where available) SES. Interaction effects by year group and gender were also investigated. In pre-specified sensitivity analyses, models were also run: with England data only (the country with largest number of data points); with data points excluded for when a survey was conducted at a different time of year (eg, SALSUS 2002 and 2006); and with survey weights applied. The extent of non-response was deemed to be sufficiently trivial for the analysis to be conducted on a complete-case basis, with data unavailable on the primary outcomes of ever and regular smoking for only 1.9% and 1.8% of pupils within the final dataset, respectively. While models were a priori assumed linear, examination of trends over time pointed towards non-linearity for some outcomes. Entry of a quadratic term to the model which allowed for structural departures from linearity changed the size and direction of odds ratios in models, revealing sensitivity to these assumptions. Quadratic models are therefore reported alongside linear models and are referred to from here on as the final models. As most trends were clearly linear from the turn of the millennium, a further post-hoc sensitivity analysis involved exclusion of the earliest time points and modelling of ‘pre-intervention’ trend from 2001 to 2010 only. All analyses were run using STATA/SE 14.2.

## Results

For primary and tertiary outcomes, data from at least one UK country were available for each of 18 time points, representing 248 324 survey respondents. For smoking attitudes, at least 15 time points were available representing 162 324 survey respondents. Across all outcomes, prevalence rates decreased over the study period (see online supplementary material tables 1-14). From 1998 to 2015, among children aged 13 and 15, the percentage of ever smokers decreased from 60% (n=3 792) to 19% (n=6 852) while regular smokers decreased from 19% (n=1 209) to 5% (n=1 618; note 2015 did not include data from England; see online supplementary tables 1 and 3, respectively). Perceptions of smoking also changed over time: the percentage of participants who reported that trying a cigarette was ‘OK’ declined from 70% (n=2 407) in 1999 to 27% (n=6 412) in 2015 (see online supplementary table 5). The percentage of young people in England reporting that it was ‘OK’ to smoke weekly declined from 36% (n=1 434) in 2003 to 14% (n=334) in 2014 (where including those who responded ‘I don’t know’ as ‘not OK’, see online supplementary table 9). With ‘I don’t know’ responses coded as ‘OK’, the percentage of participants who reported that trying a cigarette was ‘OK’ declined from 79% (n=2 859) in 1999 to 42% (n=9 904) in 2015 (see online supplementary table 7) and the percentage of participants in England who reported it was ‘OK’ to smoke weekly declined from 47% (n=1 876) in 2003 to 23% (n=554) in 2014 (see online supplementary table 10; from here on, analyses are reported with ‘I don’t know’ coded as ‘not OK’, and sensitivity analyses where ‘I don’t know’ was coded as ‘OK’ can be found in online supplementary material tables 7–8, 1 and 18). Between 1998 and 2015, ever cannabis use decreased from 29% (n=1 415) to 9% (n=3 052) and ever alcohol use from 79% (n= 2904) to 48% (n=16 866; see online supplementary materials tables 11–14).

10.1136/tobaccocontrol-2018-054584.supp1 Supplementary data

**Table 1:** Odds ratios of linear and quadratic models for ever smoked and regular smoking between 1998–2015 among students in England, Scotland and Wales

		Ever smoked				Regular smoking			
		Linear	P value	Quadratic	P value	Linear	P value	Quadratic	P value
Whole sample (n=242 855; 243 111)	Year	0.91 (0.91 to 0.91)	<0.001	0.95 (0.93 to 0.96)	<0.001	0.93 (0.93 to 0.94)	<0.001	0.98 (0.96 to 1.00)	0.025
	Year^2^	–	–	1.00 (1.00 to 1.00)	<0.001	–	–	1.00 (1.00 to 1.00)	<0.001
	Level	0.89 (0.84 to 0.95)	<0.001	0.89 (0.84 to 0.95)	<0.001	0.79 (0.71 to 0.88)	<0.001	0.80 (0.72 to 0.90)	<0.001
	Post-slope	0.97 (0.96 to 0.99)	<0.001	1.01 (0.99 to 1.03)	0.231	0.98 (0.96 to 1.01)	0.231	1.04 (1.00 to 1.08)	0.028
Male only subgroup (n=121 879; 122 042)	Year	0.92 (0.92 to 0.93)	<0.001	0.93 (0.91 to 0.95)	<0.001	0.94 (0.94 to 0.95)	<0.001	0.95 (0.92 to 0.98)	<0.001
	Year^2^	–	–	1.00 (1.00 to 1.00)	0.621	–	–	1.00 (1.00 to 1.00)	0.583
	Level	0.88 (0.81 to 0.96)	0.005	0.88 (0.81 to 0.96)	0.005	0.83 (0.70 to 0.97)	0.022	0.83 (0.70 to 0.97)	0.023
	Post-slope	0.98 (0.95 to 1.00)	0.034	0.98 (0.95 to 1.01)	0.212	1.00 (0.96 to 1.04)	0.921	1.01 (0.96 to 1.06)	0.794
Female only subgroup (n=120 976; 121 069)	Year	0.90 (0.89 to 0.90)	<0.001	0.96 (0.94 to 0.98)	<0.001	0.92 (0.92 to 0.93)	<0.001	1.00 (0.98 to 1.03)	0.922
	Year^2^	–	–	1.00 (0.99 to 1.00)	<0.001	–	–	0.99 (0.99 to 1.00)	<0.001
	Level	0.90 (0.83 to 0.98)	0.014	0.90 (0.83 to 0.98)	0.018	0.76 (0.65 to 0.89)	<0.001	0.78 (0.67 to 0.91)	0.002
	Post–slope	0.97 (0.95 to 0.99)	0.009	1.05 (1.01 to 1.08)	0.003	0.97 (0.93 to 1.01)	0.139	1.07 (1.02 to 1.12)	0.009
13 year olds only (n=126 960; 127 100)	Year	0.89 (0.89 to 0.90)	<0.001	0.96 (0.94 to 0.98)	<0.001	0.91 (0.90 to 0.92)	<0.001	1.02 (0.98 to 1.05)	0.378
	Year^2^	–	–	1.00 (0.99 to 1.00)	<0.001	–	–	0.99 (0.99 to 1.00)	<0.001
	Level	0.82 (0.74 to 0.91)	<0.001	0.83 (0.75 to 0.92)	<0.001	0.73 (0.57 to 0.92)	0.009	0.76 (0.60 to 0.97)	0.027
	Post-slope	0.99 (0.96 to 1.01)	0.304	1.07 (1.03 to 1.10)	<0.001	1.00 (0.95 to 1.07)	0.902	1.14 (1.06 to 1.23)	<0.001
15 year olds only (n=115 895; 116 011)	Year	0.93 (0.93 to 0.93)	<0.001	0.94 (0.92 to 0.95)	<0.001	0.94 (0.93 to 0.94)	<0.001	0.97 (0.95 to 0.99)	0.003
	Year^2^	–	–	1.00 (1.00 to 1.00)	0.390	–	–	1.00 (1.00 to 1.00)	0.009
	Level	0.92 (0.85 to 0.99)	0.035	0.92 (0.85 to 0.99)	0.036	0.79 (0.70 to 0.90)	<0.001	0.80 (0.70 to 0.90)	<0.001
	Post-slope	0.96 (0.94 to 0.98)	<0.001	0.96 (0.94 to 0.99)	0.012	0.98 (0.95 to 1.01)	0.240	1.01 (0.97 to 1.06)	0.497

**Table 2:** Odds ratios of linear and quadratic models for smoking attitudes among students in England and Scotland (trying smoking is ‘OK’, from 1999 to 2015) and England only (smoking weekly is ‘OK’, from 2003 to 2014)

		Trying smoking is OK* (don’t know=not OK)				Smoking weekly is OK† (don’t know=not OK)			
		Linear	P value	Quadratic	P value	Linear	P value	Quadratic	P value
Whole sample (n=165 199; 35 890)	Year	0.91 (0.91 to 0.92)	<0.001	0.87 (0.85 to 0.89)	<0.001	0.91 (0.90 to 0.92)	<0.001	0.72 (0.65 to 0.81)	<0.001
	Year^2^	–	–	1.00 (1.00 to 1.00)	<0.001	–	–	1.01 (1.01 to 1.02)	<0.001
	Level	1.03 (0.97 to 1.10)	0.330	1.05 (0.98 to 1.12)	0.143	1.15 (1.00 to 1.32)	0.045	1.10 (0.96 to 1.27)	0.152
	Post-slope	0.92 (0.90 to 0.93)	<0.001	0.88 (0.86 to 0.90)	<0.001	0.95 (0.90 to 1.00)	0.032	0.82 (0.75 to 0.89)	<0.001
Male only subgroup (n=82 270; 18 042)	Year	0.92 (0.91 to 0.93)	<0.001	0.84 (0.81 to 0.87)	<0.001	0.92 (0.91 to 0.94)	<0.001	0.67 (0.57 to 0.79)	<0.001
	Year^2^	–	–	1.01 (1.00 to 1.01)	<0.001	–	–	1.02 (1.01 to 1.03)	<0.001
	Level	1.08 (0.99 to 1.18)	0.090	1.11 (1.01 to 1.22)	0.024	1.33 (1.10 to 1.62)	0.004	1.27 (1.04 to 1.54)	0.020
	Post-slope	0.91 (0.89 to 0.93)	<0.001	0.84 (0.81 to 0.88)	<0.001	0.88 (0.82 to 0.94)	<0.001	0.71 (0.62 to 0.81)	<0.001
Female only subgroup (n=82 929; 17 848)	Year	0.90 (0.90 to 0.91)	<0.001	0.89 (0.86 to 0.93)	<0.001	0.90 (0.88 to 0.91)	<0.001	0.77 (0.66 to 0.91)	0.001
	Year^2^	–	–	1.00 (1.00 to 1.00)	0.492	–	–	1.01 (1.00 to 1.02)	0.067
	Level	0.98 (0.90 to 1.07)	0.718	0.99 (0.90 to 1.08)	0.789	1.02 (0.84 to 1.22)	0.866	0.99 (0.82 to 1.19)	0.917
	Post-slope	0.93 (0.91 to 0.95)	<0.001	0.92 (0.88 to 0.95)	<0.001	1.01 (0.95 to 1.08)	0.720	0.92 (0.81 to 1.04)	0.180
13 year olds only (n=85 713; 18 721)	Year	0.90 (0.89 to 0.91)	<0.001	0.89 (0.86 to 0.92)	<0.001	0.90 (0.89 to 0.92)	<0.001	0.78 (0.65 to 0.94)	0.007
	Year^2^	–	–	1.00 (1.00 to 1.00)	0.459	–	–	1.01 (1.00 to 1.02)	0.107
	Level	0.95 (0.86 to 1.05)	0.321	0.95 (0.87 to 1.05)	0.349	1.22 (0.97 to 1.53)	0.086	1.19 (0.94 to 1.49)	0.145
	Post-slope	0.94 (0.92 to 0.97)	<0.001	0.93 (0.90 to 0.97)	<0.001	0.93 (0.85 to 1.01)	0.092	0.84 (0.73 to 0.98)	0.022
15 year olds only (n=79 486; 17 169)	Year	0.93 (0.92 to 0.94)	<0.001	0.86 (0.83 to 0.89)	<0.001	0.91 (0.90 to 0.93)	<0.001	0.70 (0.60 to 0.80)	<0.001
	Year^2^	–	–	1.00 (1.00 to 1.01)	<0.001	–	–	1.01 (1.01 to 1.02)	<0.001
	Level	1.09 (1.01 to 1.19)	0.034	1.13 (1.04 to 1.23)	0.006	1.11 (0.94 to 1.32)	0.218	1.06 (0.90 to 1.26)	0.473
	Post-slope	0.89 (0.87 to 0.91)	<0.001	0.83 (0.80 to 0.86)	<0.001	0.96 (0.90 to 1.02)	0.144	0.80 (0.71 to 0.89)	<0.001

*Available for England and Scotland only.

†Available for England only.

**Table 3:** Odds ratios of linear and quadratic models for ever drunk alcohol and ever cannabis use between 1998–2015 for England, Scotland and Wales

		Ever drunk alcohol				Ever used cannabis			
		Linear	P value	Quadratic	P value	Linear	P value	Quadratic	P value
Whole sample (n=239 190; 239 457)	Year	0.91 (0.90 to 0.91)	<0.001	1.10 (1.08 to 1.12)	<0.001	0.92 (0.92 to 0.92)	<0.001	1.08 (1.07 to 1.10)	<0.001
	Year^2^	–	–	0.99 (0.99 to 0.99)	<0.001	–	–	0.99 (0.99 to 0.99)	<0.001
	Level	0.90 (0.85 to 0.95)	<0.001	0.85 (0.81 to 0.90)	<0.001	0.98 (0.90 to 1.07)	0.661	1.01 (0.93 to 1.10)	0.761
	Post-slope	0.96 (0.94 to 0.97)	<0.001	1.17 (1.14 to 1.19)	<0.001	1.00 (0.98 to 1.03)	0.667	1.21 (1.18 to 1.25)	<0.001
Male only subgroup (n=119 989; 120 025)	Year	0.91 (0.90 to 0.91)	<0.001	1.06 (1.03 to 1.09)	<0.001	0.93 (0.92 to 0.93)	<0.001	1.06 (1.04 to 1.09)	<0.001
	Year^2^	–	–	0.99 (0.99 to 0.99)	<0.001	–	–	0.99 (0.99 to 0.99)	<0.001
	Level	0.89 (0.82 to 0.96)	0.004	0.85 (0.79 to 0.92)	<0.001	0.92 (0.82 to 1.04)	0.185	0.94 (0.84 to 1.06)	0.337
	Post–slope	0.96 (0.94 to 0.98)	<0.001	1.12 (1.09 to 1.16)	<0.001	1.02 (0.99 to 1.05)	0.175	1.20 (1.15 to 1.24)	<0.001
Female only subgroup (n=119 201; 119 432)	Year	0.90 (0.90 to 0.91)	<0.001	1.15 (1.12 to 1.18)	<0.001	0.92 (0.91 to 0.92)	<0.001	1.11 (1.08 to 1.14)	<0.001
	Year^2^	–	–	0.99 (0.98 to 0.99)	<0.001	–	–	0.99 (0.99 to 0.99)	<0.001
	Level	0.91 (0.84 to 0.98)	0.016	0.85 (0.79 to 0.92)	<0.001	1.06 (0.93 to 1.20)	0.396	1.11 (0.98 to 1.26)	0.110
	Post–slope	0.96 (0.94 to 0.97)	<0.001	1.22 (1.18 to 1.26)	<0.001	0.99 (0.95 to 1.02)	0.359	1.23 (1.17 to 1.28)	<0.001
13 year olds only (n=124 842; 123 608)	Year	0.90 (0.90 to 0.90)	<0.001	1.11 (1.08 to 1.13)	<0.001	0.90 (0.89 to 0.90)	<0.001	1.16 (1.12 to 1.21)	<0.001
	Year^2^	–	–	0.99 (0.99 to 0.99)	<0.001	–	–	0.98 (0.98 to 0.99)	<0.001
	Level	0.88 (0.82 to 0.95)	0.001	0.84 (0.78 to 0.91)	<0.001	1.07 (0.89 to 1.28)	0.480	1.14 (0.95 to 1.37)	0.165
	Post–slope	0.95 (0.93 to 0.97)	<0.001	1.17 (1.14 to 1.21)	<0.001	0.99 (0.95 to 1.04)	0.746	1.34 (1.26 to 1.42)	<0.001
15 year olds only (n=114 348; 115 849)	Year	0.92 (0.91 to 0.92)	<0.001	1.08 (1.05 to 1.11)	<0.001	0.93 (0.92 to 0.93)	<0.001	1.06 (1.04 to 1.08)	<0.001
	Year^2^	–	–	0.99 (0.99 to 0.99)	<0.001	–	–	0.99 (0.99 to 0.99)	<0.001
	Level	0.92 (0.84 to 1.00)	0.049	0.87 (0.80 to 0.95)	0.002	0.96 (0.87 to 1.06)	0.432	0.99 (0.90 to 1.09)	0.776
	Post–slope	0.96 (0.94 to 0.98)	<0.001	1.13 (1.09 to 1.17)	<0.001	1.01 (0.98 to 1.03)	0.645	1.17 (1.13 to 1.20)	<0.001

Table 1 shows model results, adjusted for covariates, for ever smoked and regular smoking (for the whole sample and subgroups based on gender and year group). Table 2 shows model results, adjusted for covariates, for smoking attitudes (for the whole sample and subgroups based on gender and year group, see online supplementary tables 15 and 16 in supplementary material for subgroup analyses by SES and for England only).

**Figure 1:**
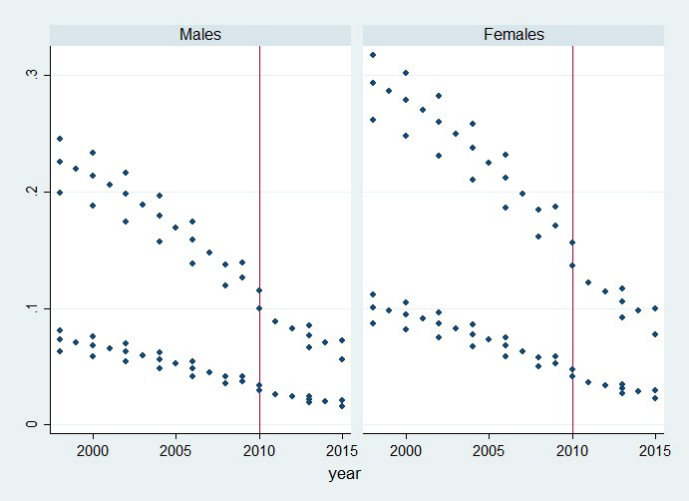
Predicted probabilities of regular smoking for males and females in England, Scotland and Wales, from logistic regression analyses (the top lines represent 15 year olds, the bottom 13 year olds).

**Figure 2:**
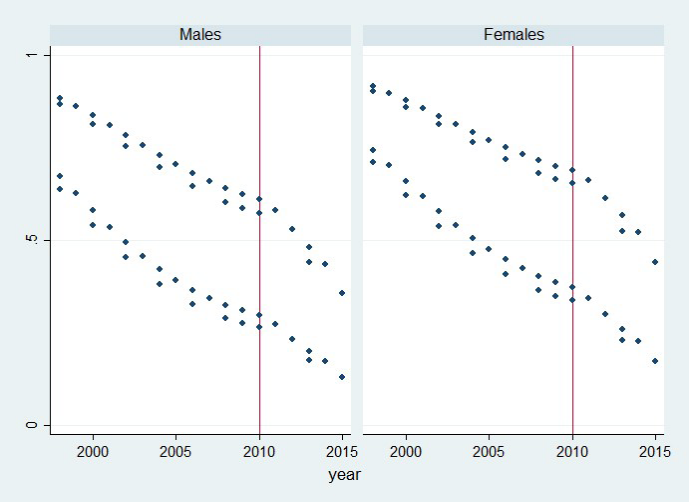
Predicted probabilities of stating that trying smoking is ‘OK’ for males and females in England and Scotland, from binary logistic regression analyses (the top lines represent 15 year olds, the bottom 13 year olds).

As indicated by the final quadratic models in table 1, for the whole sample, change in the rate of decline for ever smoking post-2010 was not significant, though a marginally significant (p=0.03) slowing in the rate of decline occurred for regular smoking. For subgroup analyses, the slowing decline in regular smoking post-2010 was limited to groups for whom rates had declined rapidly before 2010 (ie, females and 13 year olds, see figure 1). Similarly, there was a significant slowing in the rate of decline post-2010 among these subgroups for ever smoked, though a significant increase in the rate of decline for 15 year olds (see online figure 1 supplementary material). For smoking attitudes, there was consistent evidence across all subgroups of an increased rate of decline in the percentage of young people saying that trying smoking is ‘OK’ and weekly smoking is ‘OK’, except for subgroup analyses of attitudes of smoking weekly for females (see figure 2 and table 2).

For ever and regular smoking, there was a significant reduction in prevalence at the intervention point (referred to as ‘level’ in the table, and from here on) for the whole sample and all subgroups. Changes in trend were robust to pre-specified sensitivity analyses, although some England-only models differed slightly from the whole group models (see online supplementary tables 15 and 16). In post-hoc sensitivity analyses modelling trends from 2001 to account for non-linearity (not shown in tables), the statistical evidence for a change in trend for regular smoking weakened (OR 0.99, CI 0.94 to 1.03), and magnitude for change in trend lowered for ever cannabis use (OR 1.05, CI 1.01 to 1.09) and alcohol use (OR 1.05, CI 1.02 to 1.08) though remaining significant, although other findings were not materially altered. For sensitivity analyses of smoking attitudes (trying smoking is ‘OK’) not including years 1999 and 2001, no differences in results were found for change in decline (OR 0.85, CI 0.81 to 0.88; p<0.001), although the level became significant (OR 1.08, CI 1.01 to 1.16; p=0.030).

Inclusion of time*gender interaction terms showed that for all outcomes (with the exception of alcohol use) changes in the secular decline over time was significantly greater for females than for males (see online supplementary table 20). For gender, there was no significant effect modification for level or post-slope terms, except for attitudes towards smoking weekly where the rate of decline increased at a significantly faster rate for males than for females (though with a significant increase in trend for males). Inclusion of time*year group interaction terms showed that for all outcomes (with the exception of attitudes towards smoking weekly), the changes in the secular decline over time was greater for 13 year olds than for 15 year olds. Change in level post-2010 was more negative among 13 year olds for ever smoked and ever alcohol use. For post-slope terms, declines in prevalence for ever smoked, regular smoking and positive attitudes towards trying smoking were greater for 15 year olds than 13 year olds.

Decreases in rates of decline post-2010 were observed for alcohol use and cannabis and in greater magnitude than change in regular smoking (OR 1.17, CI 1.14 to 1.19, and OR 1.21, CI 1.18 to 1.25, respectively; see table 3). These were generally consistent across all subgroups (see online supplementary table 19 as well as figures 5-6 in supplementary material).

## Discussion

This study is the first to test whether proliferation of e-cigarettes during a period of limited regulation led to changes in smoking trajectories *as well as* smoking attitudes among young people. Our results provide little evidence that renormalisation of smoking occurred during this period. The rate of decline for ever smoking prevalence did not slow. While decreases for regular smoking did slow, this was specific to groups where the level of decline before 2010 was greatest, possibly reflecting a floor effect in the data. Slowing declines were also found, to a greater magnitude, for cannabis and alcohol use, suggesting change in trend was not unique to tobacco use, but reflected wider changes in youth substance use trajectories. What is more, positive perceptions of smoking attitudes declined at a *faster rate* following the proliferation of e-cigarettes, suggesting that attitudes towards smoking hardened while e-cigarettes were emerging rather than softening, as would be expected were smoking becoming renormalised. These findings are consistent with a previous study in the USA that found little change in smoking trends among adolescents during a period of growth in e-cigarette use.^27^ Our study is, however, unique in that it is the first to test these changes in the UK population, and to understand them in the context of broader substance use trajectories. It is the first internationally to test the renormalisation hypothesis by examining changes in trends for youth attitudes toward smoking. Although it is unclear to what extent our findings can generalise to other countries, the UK is often referred to as a country comparable to the USA with regards to the tobacco epidemic.^23^

This study benefits from the use of a large, nationally representative sample of school-age children from England, Scotland and Wales, covering a long time period (17 years). It also benefited from investigating smoking attitudes, contributing to understanding underlying theoretical mechanisms of renormalisation hypothesis, and locating changes in smoking within the context of wider youth substance use trajectories to assess whether or not findings were unique to smoking outcomes. Nevertheless, it does suffer some substantial limitations. Survey intervals and the methods used varied. While all surveys used two-stage cluster sampling, recruiting schools and then pupils, the absence of school identifiers within some datasets precluded adjustment for clustering. Smoking typically exhibits a moderate to high degree of intra-cluster correlation.^52^ Hence, adjusting for clustering would likely have led to a change in trends, such as that for smoking regularly where significance was borderline (p=0.03), becoming non-significant. It would likely have had less of an impact on results for smoking attitudes, which had p values typically below 0.001. Robust country-specific analyses were only possible for England as this country provided the most frequently occurring data points before and after the intervention time point. Stratification by SES based on free school meal entitlement was only possible for England and Scotland data, as survey data from Wales did not contain an equivalent indicator of SES, and findings from these subgroups are presented with caution. Events other than the increased use of e-cigarettes might have contributed to the increased decline in positive smoking attitudes observed in the current study, and causality cannot be asserted. The fact that estimates are available only on an annual or biennial basis limits our ability to understand covariance between e-cigarettes and tobacco use over time.

Nevertheless, the study has important implications. It demonstrates the success of public health efforts in reducing smoking among young people. With the average prevalence levels of ever smokers having decreased by nearly 40 percentage points for adolescents within two decades, it is no surprise many fear a reversal in this progress. However, given the limited evidence for the renormalisation of youth smoking, it is perhaps unhelpful for policy on e-cigarette regulation to be justified on the sole basis that they renormalise smoking.^20^ Some evidence from animal models suggests that nicotine use during adolescence can inhibit brain development. Because of this, use of e-cigarettes among young people has been described as a potential concern in its own right. While evidence to date suggests that regular use among non-smokers is rare,^17^ continued conflation with the normalisation of tobacco may be an unhelpful distraction from the need to consider whether youth e-cigarette use does become a potential problem in isolation from its links to tobacco.

Understanding young people’s perceptions of e-cigarettes, the ways in which they are viewed as similar or different to cigarettes, and how these vary according to regulatory frameworks, is an important direction for future research. It remains to be seen whether trajectories of e-cigarette use, smoking and smoking attitudes will change (positively or negatively) as a result of increased e-cigarette regulations such as the marketing restrictions and product labelling brought in by the EU TPD. Within the UK, while regulatory frameworks have to date been similar, Welsh and Scottish governments^53^ have pursued (but not yet implemented) more restrictive regulatory frameworks than England.^54^ Wales was the only country whose government attempted (unsuccessfully) to ban vaping in public places, while Scotland is considering further restrictions on marketing of e-cigarettes. Future research focusing on how divergences in policy impact young people’s use of and attitudes toward tobacco, and e-cigarettes, would further enhance our understandings of these issues.

While this policy landscape is shifting, so are the products themselves. E-cigarettes have been described as mimicking behavioural aspects of smoking; as discussed, the renormalisation hypothesis is premised on the assumption that cigarettes and e-cigarettes are viewed as similar to each other.^23^ However, e-cigarettes have changed substantially over time and now resemble traditional cigarettes less than early ‘cig-a-like’ models, which may decrease perceived similarity. Saebo and Scheffels^23^ state that the normalisation of e-cigarettes can occur during the simultaneous continued de-normalisation of cigarette use, and this appears to be reflected in the findings reported here. However, newer products entering the market have been described by some as showing particular popularity among young people in the USA.^55^ Hence, while neither widespread regular youth vaping, nor the renormalisation of smoking, appear to have occurred during the period investigated here, ongoing monitoring of young people’s e-cigarette use, and links to smoking, remains a public health priority.

**What this paper adds**

**What is already known about this topic**

- E-cigarette experimentation is increasing among young people who have not previously used tobacco, leading to fears that e-cigarettes may renormalise smoking.
- However, e-cigarette experimentation is not translating into regular e-cigarette use, and smoking rates among young people continue to fall.
- It has not been tested whether the proliferation of e-cigarettes has renormalised, or displaced, smoking behaviour and smoking attitudes among young people.

**What this study adds**

- While the rate of decline for regular smoking did marginally slow between 2011–2015, this was also found for cannabis and alcohol use. Furthermore, the decline in the perceived acceptability of smoking behaviour accelerated during this period.
- Our findings do not support the hypothesis that e-cigarettes renormalised youth smoking during a period of growing but largely unregulated use in the UK.
