# Supplementary material for: Have e-cigarettes renormalised or displaced youth smoking? Results of a segmented regression analysis of repeated cross sectional survey data in England, Scotland and Wales
Source: Tob Control. 2019 Apr 1;29(2):207–16. doi: 10.1136/tobaccocontrol-2018-054584 (PMC7036293; doi:10.1136/tobaccocontrol-2018-054584)

Supplementary Material

Additional sensitivity analyses

The Scottish Adolescent Lifestyle and Substance Use Survey (SALSUS) also reported the Scottish Index of Multiple Deprivation (SIMD) in addition to information about student's receiving free school meals. SIMD was reported in quintiles (quintiles 1-3 = most deprived; quintiles 4-5 = least deprived) from years 2006-2015. This was also used as an indicator for high and low SES and used for sensitivity analyses. As analyses for Scotland-only data include fewer data points, these are presented with caution.

For the year 2000 in HBSC, a binary yes/no question was used as this pre-dated the start of the items used in the following years. Additionally, there was no cannabis variable and alcohol variables remained unclear. For analyses, leaving that year in or out of the main models for smoking behaviours, cannabis and alcohol use, made no difference to the results.

Prevalence rates and CI (95%) for study outcomes over time

Table 1: Prevalence and CI (95%) of ever smokers among students between 1998-2015, by year and by country, in England, Scotland and Wales

		95% CI			95% CI			95% CI			95% CI	
Year	**All** (%)	Low	High	**England** (%)	Low	High	**Scotland** (%)	Low	High	**Wales** (%)	Low	High
1998	59.8	58.5	61.0	60.2	58.0	62.4	61.9	59.6	64.2	58.0	56.1	59.9
1999	54.2	52.6	55.9	54.2	52.6	55.9	-	-	-	-	-	-
2000	52.8	51.6	53.9	52.2	50.4	54.1	50.1	48.1	52.2	56.1	54.1	58.2
2001	53.1	51.5	54.8	53.1	51.5	54.8	-	-	-	-	-	-
2002	50.0	49.4	50.6	50.1	48.5	51.7	50.1	49.5	50.8	48.4	46.4	50.4
2003	52.4	50.8	54.0	52.4	50.8	54.0	-	-	-	-	-	-
2004	47.3	46.4	48.1	48.2	46.6	49.9	47.4	46.2	48.6	45.6	43.7	47.5
2005	50.7	49.0	52.4	50.7	49.0	52.4	-	-	-	-	-	-
2006	39.8	39.2	40.4	47.7	45.9	49.5	38.6	37.9	39.2	40.6	38.8	42.4
2007	43.1	41.3	44.9	43.1	41.3	44.9	-	-	-	-	-	-
2008	37.3	36.5	38.2	40.9	39.2	42.7	36.2	35.3	37.2	-	-	-
2009	32.7	31.5	33.8	37.8	36.1	39.6	-	-	-	28.2	26.8	29.8
2010	32.4	31.9	32.8	35.5	33.7	37.2	32.1	31.7	32.6	-	-	-
2011	32.9	31.1	34.8	32.9	31.1	34.8	-	-	-	-	-	-
2012	31.6	30.0	33.3	31.6	30.0	33.3	-	-	-	-	-	-
2013	22.6	22.2	23.1	27.3	25.3	29.2	22.5	22.1	23.0	21.1	19.7	22.5
2014	24.8	23.1	26.6	24.8	23.1	26.6	-	-	-	-	-	-
2015	19.1	18.7	19.5	-	-	-	20.0	19.5	20.5	17.1	16.4	17.8

Table 2: Prevalence and CI (95%) of ever smokers among students between 1998-2015, by gender and school year, in England, Scotland and Wales

		95% CI			95% CI			95% CI			95% CI	
Year	**Males** (%)	Low	High	**Females** (%)	Low	High	**13 year olds** (%)	Low	High	**15 year olds** (%)	Low	High
1998	54.1	52.3	55.8	65.5	63.8	67.1	50.3	48.3	52.2	66.2	64.7	67.7
1999	50.8	48.5	53.1	58.0	55.7	60.4	43.4	41.1	45.6	66.0	63.8	68.3
2000	47.6	46.0	49.2	58.2	56.6	59.9	42.6	41.0	44.2	63.8	62.2	65.4
2001	49.8	47.5	52.1	56.5	54.2	58.8	43.7	41.5	46.0	64.2	61.8	66.5
2002	44.8	44.0	45.6	55.2	54.4	56.0	42.4	41.7	43.2	58.8	58.0	59.7
2003	48.7	46.5	50.9	56.2	54.0	58.4	42.5	40.4	44.6	63.5	61.2	65.6
2004	41.7	40.5	42.9	52.8	51.6	54.1	37.6	36.5	38.8	57.9	56.6	59.1
2005	46.1	43.8	48.4	55.5	53.2	57.9	39.9	37.7	42.1	63.7	61.3	66.0
2006	35.4	34.6	36.1	44.1	43.3	45.0	29.5	28.8	30.2	50.7	49.9	51.5
2007	37.6	35.2	40.1	48.8	46.2	51.3	31.7	29.4	34.1	55.0	52.5	57.6
2008	34.8	33.7	36.0	39.8	38.6	41.0	26.4	25.3	27.4	49.6	48.4	50.9
2009	29.6	28.0	31.1	35.9	34.2	37.6	21.4	20.1	22.8	45.2	43.4	47.0
2010	30.7	30.1	31.4	34.0	33.3	34.7	20.9	20.3	21.4	44.8	44.0	45.5
2011	30.9	28.3	33.6	34.7	32.1	37.3	21.8	19.6	24.2	44.7	41.9	47.6
2012	31.3	29.0	33.8	31.9	29.6	34.4	20.3	18.3	22.3	44.1	41.5	46.7
2013	21.3	20.7	21.9	24.0	23.4	24.6	12.2	11.8	12.7	33.8	33.1	34.5
2014	23.4	21.0	25.8	26.3	23.8	28.9	15.4	13.4	17.5	35.0	32.3	37.9
2015	18.2	17.7	18.8	19.8	19.2	20.3	10.9	10.4	11.3	28.8	28.2	29.5

Table 3: Prevalence and CI (95%) of regular smokers among students between 1998-2015, by year and by country, in England, Scotland and Wales

		95% CI			95% CI			95% CI			95% CI	
Year	**All** (%)	Low	High	**England** (%)	Low	High	**Scotland** (%)	Low	High	**Wales** (%)	Low	High
1998	19.0	18.1	20.0	19.5	17.7	21.3	18.7	16.9	20.6	18.9	17.4	20.4
1999	14.7	13.6	15.9	14.7	13.6	15.9	-	-	-	-	-	-
2000	15.4	14.6	16.3	16.0	14.7	17.4	13.1	11.7	14.5	17.2	15.6	18.8
2001	14.7	13.6	16.0	14.7	13.6	16.0	-	-	-	-	-	-
2002	13.9	13.5	14.3	16.0	14.8	17.2	13.2	12.8	13.7	16.2	14.8	17.7
2003	14.3	13.2	15.4	14.3	13.2	15.4	-	-	-	-	-	-
2004	13.5	12.9	14.1	13.6	12.6	14.8	12.4	11.6	13.2	16.1	14.7	17.5
2005	13.3	12.2	14.5	13.3	12.2	14.5	-	-	-	-	-	-
2006	10.2	9.9	10.6	13.3	12.2	14.6	9.4	9.0	9.8	13.1	11.9	14.4
2007	10.0	9.0	11.1	10.0	9.0	11.1	-	-	-	-	-	-
2008	9.2	8.7	9.7	9.9	8.9	11.0	9.0	8.4	9.5	-	-	-
2009	8.9	8.2	9.6	9.4	8.4	10.5	-	-	-	8.4	7.5	9.4
2010	7.9	7.6	8.1	8.6	7.6	9.7	7.8	7.5	8.1	-	-	-
2011	6.7	5.7	7.7	6.7	5.7	7.7	-	-	-	-	-	-
2012	6.6	5.8	7.6	6.6	5.8	7.6	-	-	-	-	-	-
2013	5.1	4.9	5.3	5.0	4.1	6.1	5.1	4.8	5.3	5.4	4.7	6.2
2014	5.5	4.7	6.5	5.5	4.7	6.5	-	-	-	-	-	-
2015	4.5	4.3	4.7	-	-	-	4.1	3.8	4.3	5.5	5.1	6.0

Table 4: Prevalence and CI (95%) of regular smokers among students between 1998-2015, by gender and school year, in England, Scotland and Wales

		95% CI			95% CI			95% CI			95% CI	
Year	**Males** (%)	Low	High	**Females** (%)	Low	High	**13 year olds** (%)	Low	High	**15 year olds** (%)	Low	High
1998	15.5	14.2	16.8	22.6	21.1	24.1	10.5	9.4	11.8	24.7	23.4	26.1
1999	13.1	11.6	14.7	16.5	14.7	18.3	6.7	5.6	7.9	23.4	21.4	25.4
2000	12.3	11.3	13.4	18.7	17.5	20.1	8.5	7.6	9.4	23.0	21.6	24.4
2001	12.4	10.9	14.0	17.1	15.4	19.0	8.6	7.4	9.9	22.0	20.0	24.1
2002	11.2	10.7	11.7	16.5	15.9	17.1	8.2	7.8	8.6	20.5	19.8	21.2
2003	11.0	9.7	12.5	17.6	15.9	19.3	7.2	6.1	8.4	22.1	20.3	24.1
2004	10.4	9.7	11.2	16.6	15.7	17.5	7.1	6.5	7.7	20.5	19.5	21.6
2005	10.8	9.4	12.3	15.9	14.2	17.7	6.8	5.8	8.1	21.0	19.1	23.1
2006	8.2	7.7	8.6	12.2	11.7	12.8	4.8	4.5	5.2	15.9	15.3	16.5
2007	8.0	6.7	9.5	12.0	10.4	13.8	4.4	3.4	5.5	15.9	14.1	17.9
2008	8.1	7.4	8.8	10.3	9.5	11.0	4.2	3.7	4.7	14.8	13.9	15.7
2009	7.4	6.5	8.3	10.4	9.4	11.5	4.1	3.4	4.8	14.2	13.0	15.5
2010	7.1	6.7	7.4	8.6	8.2	9.0	3.1	2.9	3.4	13.0	12.5	13.4
2011	6.4	5.1	8.0	6.9	5.6	8.4	3.1	2.2	4.2	10.6	8.9	12.4
2012	6.8	5.6	8.2	6.4	5.3	7.8	2.2	1.6	3.1	11.5	9.9	13.2
2013	4.8	4.5	5.1	5.3	5.0	5.7	1.9	1.7	2.1	8.5	8.1	8.9
2014	5.0	3.9	6.4	6.1	4.8	7.6	2.5	1.7	3.5	8.9	7.3	10.7
2015	4.4	4.1	4.7	4.5	4.2	4.8	1.9	1.7	2.1	7.6	7.2	8.0

Table 5: Prevalence and CI (95%) of young people who think trying smoking is “OK” (don’t know = not OK) between 1999-2015, by year and by country, among students in England and Scotland

		95% CI			95% CI			95% CI	
Year	**All** (%)	Low	High	**England** (%)	Low	High	**Scotland** (%)	Low	High
1999	70.0	68.4	71.5	70.0	68.4	71.5	-	-	-
2000	-	-	-	-	-	-	-	-	-
2001	70.0	68.4	71.5	70.0	68.4	71.5	-	-	-
2002	-	-	-	-	-	-	-	-	-
2003	63.8	62.3	65.3	63.8	62.3	65.3	-	-	-
2004	55.8	54.2	57.5	55.8	54.2	57.5	-	-	-
2005	59.1	57.4	60.7	59.1	57.4	60.7	-	-	-
2006	53.4	52.8	54.0	51.1	49.3	52.9	53.8	53.1	54.4
2007	52.9	51.1	54.7	52.9	51.1	54.7	-	-	-
2008	47.9	47.0	48.8	48.9	47.1	50.7	47.6	46.6	48.6
2009	52.4	50.6	54.3	52.4	50.6	54.3	-	-	-
2010	45.5	45.0	46.0	48.6	46.7	50.4	45.2	44.7	45.7
2011	48.0	46.0	50.0	48.0	46.0	50.0	-	-	-
2012	43.1	41.3	44.9	43.1	41.3	44.9	-	-	-
2013	33.6	33.1	34.1	42.7	40.5	44.9	33.1	32.5	33.6
2014	35.1	33.2	37.1	35.1	33.2	37.1	-	-	-
2015	27.0	26.5	27.6	-	-	-	27.0	26.5	27.6

Table 6: Prevalence and CI (95%) of young people who think trying smoking is "OK" (don't know = not OK) between 1999-2015, by gender and school year, among students in England and Scotland

		95% CI			95% CI			95% CI			95% CI	
Year	**Males** (%)	Low	High	**Females** (%)	Low	High	**13 year olds** (%)	Low	High	**15 year olds** (%)	Low	High
1999	66.1	63.9	68.2	74.2	72.0	76.2	58.8	56.6	61.1	81.9	80.0	83.7
2000	-	-	-	-	-	-	-	-	-	-	-	-
2001	66.0	63.7	68.2	74.0	71.8	76.0	62.0	59.8	64.2	79.3	77.3	81.3
2002	-	-	-	-	-	-	-	-	-	-	-	-
2003	59.6	57.4	61.7	68.1	66.0	70.2	54.0	51.9	56.2	74.7	72.6	76.6
2004	49.6	47.3	51.9	62.4	60.1	64.7	43.4	41.2	45.7	69.8	67.6	72.0
2005	53.0	50.6	55.3	65.4	63.1	67.6	47.2	45.0	49.5	73.3	71.1	75.5
2006	48.5	47.6	49.4	58.3	56.4	59.1	39.1	38.3	40.0	68.2	67.4	69.0
2007	46.6	44.1	49.1	59.3	56.7	61.8	39.0	36.6	41.5	67.3	64.9	69.7
2008	43.5	42.2	44.7	52.1	50.9	53.4	33.8	32.7	35.0	63.3	62.1	64.6
2009	48.0	45.4	50.6	57.0	54.4	59.6	37.7	35.3	40.2	68.3	65.8	70.7
2010	42.2	41.5	43.0	48.7	48.0	49.4	29.4	28.8	30.1	62.4	61.7	63.1
2011	45.2	42.4	48.1	50.5	47.7	53.2	33.4	30.8	36.0	63.5	60.7	66.2
2012	40.0	37.5	42.6	46.2	43.6	48.8	28.5	26.3	30.8	59.0	56.4	61.6
2013	31.1	30.4	31.8	36.1	35.4	36.9	18.6	18.0	19.1	49.5	48.7	50.2
2014	30.8	28.2	33.4	39.6	36.8	42.5	20.8	18.6	23.2	50.6	47.6	53.5
2015	24.9	24.1	25.7	28.8	28.0	29.6	15.3	14.7	16.0	40.5	39.6	41.4

Table 7: Prevalence and CI (95%) of young people who think trying smoking is "OK" (don't know = OK) between 1999-2015, by year and by country, among students in England and Scotland

		95% CI			95% CI			95% CI	
Year	**All** (%)	Low	High	**England** (%)	Low	High	**Scotland** (%)	Low	High
1999	79.5	78.2	80.8	79.5	78.2	80.8	-	-	-
2000	-	-	-	-	-	-	-	-	-
2001	78.9	77.5	80.2	78.9	77.5	80.2	-	-	-
2002	-	-	-	-	-	-	-	-	-
2003	73.8	72.4	75.1	73.8	72.4	75.1	-	-	-
2004	65.6	64.0	67.1	65.6	64.0	67.1	-	-	-
2005	69.8	68.2	71.3	69.8	68.2	71.3	-	-	-
2006	63.4	62.7	64.0	61.9	60.2	63.6	63.6	62.9	64.2
2007	63.6	61.8	65.3	63.6	61.8	65.3	-	-	-
2008	59.1	58.2	59.9	59.4	57.6	61.1	59.0	58.0	60.0
2009	63.3	61.5	65.0	63.3	61.5	65.0	-	-	-
2010	56.7	56.2	57.2	61.0	59.2	62.8	56.4	55.9	56.9
2011	60.4	58.5	62.4	60.4	58.5	62.4	-	-	-
2012	53.9	52.1	55.7	53.9	52.1	55.7	-	-	-
2013	46.0	45.5	46.5	53.6	51.4	55.8	45.5	45.0	46.0
2014	46.8	44.8	48.8	46.8	44.8	48.8	-	-	-
2015	41.7	41.1	42.4	-	-	-	41.7	41.1	42.4

Table 8: Prevalence and CI (95%) of young people who think trying smoking is "OK" (don't know = OK) between 1999-2015, by gender and school year, among students in England and Scotland

		95% CI			95% CI			95% CI			95% CI	
Year	**Males** (%)	Low	High	**Females** (%)	Low	High	**13 year olds** (%)	Low	High	**15 year olds** (%)	Low	High
1999	75.7	73.7	77.6	83.7	81.9	85.4	71.6	69.4	73.6	88.1	86.5	89.6
2000	-	-	-	-	-	-	-	-	-	-	-	-
2001	75.7	73.7	77.7	82.0	80.2	83.8	72.8	70.7	74.8	86.1	84.3	87.7
2002	-	-	-	-	-	-	-	-	-	-	-	-
2003	69.7	67.6	71.7	78.0	76.1	79.8	66.8	64.7	68.8	81.6	79.7	83.3
2004	60.3	58.0	62.5	71.1	68.9	73.2	55.8	53.6	58.0	76.5	74.5	78.5
2005	65.0	62.8	67.2	74.7	72.5	76.7	60.5	58.2	62.6	81.0	79.0	82.9
2006	58.9	58.0	59.7	67.7	66.9	68.6	50.9	50.0	51.8	76.2	75.5	77.0
2007	58.0	55.4	60.4	69.4	67.0	71.7	52.3	49.7	54.8	75.4	73.1	77.6
2008	55.0	53.8	56.3	62.9	61.7	64.1	47.3	46.1	48.5	72.0	70.8	73.1
2009	59.5	56.9	62.0	67.3	64.8	69.7	50.7	48.2	53.3	76.8	74.5	79.0
2010	54.5	53.8	55.2	58.9	58.2	59.6	42.9	42.2	43.6	71.3	70.7	72.0
2011	58.0	55.1	60.8	62.6	59.9	65.2	48.5	45.7	51.3	73.1	70.5	75.5
2012	51.4	48.8	54.0	56.4	53.9	58.9	40.9	38.4	43.4	68.1	65.6	70.5
2013	43.7	43.0	44.5	48.2	47.4	48.9	31.9	31.2	32.6	60.8	60.1	61.5
2014	41.9	39.1	44.7	52.0	49.1	54.8	34.8	32.1	37.5	59.8	56.9	62.7
2015	40.0	39.1	40.9	43.1	42.2	44.0	30.1	29.3	30.9	55.2	54.2	56.1

Table 9: Prevalence and CI (95%) of students in England who think smoking weekly is “OK” (don’t know = not OK) between 2003-2014, by gender and year group

		95% CI			95% CI			95% CI			95%			95%	
Year	**All** (%)	Low	High	**Males** (%)	Low	High	**Females** (%)	Low	High	**13 year olds** (%)	Low	High	**15 year olds** (%)	Low	High
2003	36.2	34.7	37.7	32.8	30.7	34.9	39.7	37.5	41.9	24.3	22.5	26.2	49.4	47.2	51.7
2004	28.3	26.9	29.8	24.7	22.8	26.8	32.1	29.9	34.3	17.4	15.7	19.1	40.7	38.3	43.1
2005	30.8	29.3	32.4	26.9	24.8	29.0	34.9	32.6	37.2	21.4	19.6	23.3	42.1	39.6	44.5
2006	25.7	24.1	27.2	23.1	21.0	25.4	28.0	25.8	30.2	15.5	13.8	17.4	36.4	33.9	38.8
2007	27.3	25.7	28.9	24.2	22.1	26.4	30.5	28.2	32.9	16.4	14.6	18.4	38.7	36.2	41.2
2008	21.2	19.7	22.7	18.6	16.7	20.7	23.8	21.6	26.1	14.2	12.5	16.1	28.5	26.2	30.9
2009	25.0	23.5	26.6	24.5	22.3	26.7	25.6	23.4	27.9	14.2	12.4	16.0	36.7	34.2	39.3
2010	22.3	20.8	23.9	21.4	19.3	23.7	23.2	21.0	25.5	13.2	11.6	15.1	32.4	29.9	34.9
2011	20.4	18.8	22.0	20.3	18.1	22.8	20.4	18.3	22.7	11.9	10.2	13.8	29.3	26.8	32.0
2012	19.6	18.2	21.1	19.1	17.1	21.2	20.2	18.2	22.3	12.8	11.1	14.5	27.1	24.8	29.5
2013	17.5	15.9	19.2	16.6	14.4	18.9	18.6	16.1	21.2	8.3	6.7	10.1	27.1	24.4	30.0
2014	13.9	12.6	15.4	12.0	10.2	14.0	15.9	13.9	18.1	8.0	6.6	9.7	20.3	18.0	22.8

Table 10: Prevalence and CI (95%) of students in England who think smoking weekly is "OK" (don't know = OK) between 2003-2014, by gender and year group

		95% CI			95% CI			95% CI			95%			95%	
Year	**All** (%)	Low	High	**Males** (%)	Low	High	**Females** (%)	Low	High	**13 year olds** (%)	Low	High	**15 year olds** (%)	Low	High
2003	47.3	45.8	48.9	43.7	41.5	45.9	51.1	48.9	53.4	36.7	34.6	38.8	59.2	56.9	61.4
2004	38.0	36.4	39.6	33.9	31.8	36.1	42.1	39.8	44.5	27.5	25.5	29.6	49.7	47.3	52.1
2005	41.7	40.1	43.4	37.1	34.8	39.4	46.5	44.1	48.9	32.0	29.9	34.1	53.4	50.9	55.9
2006	36.0	34.3	37.7	33.9	31.5	36.3	37.9	35.5	40.3	26.3	24.2	28.6	46.1	43.6	48.7
2007	38.7	36.9	40.4	35.6	33.2	38.0	41.8	39.3	44.4	28.7	26.4	31.0	49.1	46.5	51.6
2008	30.6	28.9	32.2	27.9	25.6	30.2	33.3	30.9	35.8	23.0	20.9	25.2	38.5	36.0	41.1
2009	35.7	34.0	37.5	35.1	32.6	37.5	36.4	33.9	38.9	24.7	22.6	26.9	47.5	44.9	50.1
2010	33.5	31.8	35.3	33.0	30.5	35.5	34.1	31.6	36.6	25.7	23.5	28.0	42.3	39.6	45.0
2011	31.2	29.4	33.1	30.9	28.2	33.6	31.6	29.0	34.1	22.8	20.5	25.2	40.2	37.4	43.0
2012	28.5	26.9	30.2	28.2	25.9	30.6	28.8	26.5	31.1	21.2	19.2	23.3	36.4	33.9	39.0
2013	25.6	23.7	27.5	23.9	21.4	26.2	27.5	24.7	30.5	16.3	14.1	18.7	35.3	32.3	38.3
2014	23.1	21.4	24.8	20.1	17.9	22.5	26.2	23.7	28.8	16.5	14.5	18.7	30.2	27.6	33.0

Table 11: Prevalence and CI (95%) of ever cannabis use between 1998-2015, by year and by country, among students in England, Scotland and Wales

		95% CI			95% CI			95% CI			95% CI	
Year	**All** (%)	Low	High	**England** (%)	Low	High	**Scotland** (%)	Low	High	**Wales** (%)	Low	High
1998	28.5	27.2	29.7	24.7	22.8	26.6	27.0	24.9	29.2	36.4	33.7	39.1
1999	21.4	20.1	22.8	21.4	20.1	22.8	-	-	-	-	-	-
2000	21.0	20.0	21.9	19.8	18.3	21.3	19.8	18.3	21.5	23.5	21.8	25.3
2001	25.0	23.6	26.5	25.0	23.6	26.5	-	-	-	-	-	-
2002	23.6	23.1	24.1	24.6	23.3	26.1	22.9	22.3	23.4	34.2	31.5	37.0
2003	25.9	24.5	27.3	25.9	24.5	27.3	-	-	-	-	-	-
2004	23.6	22.9	24.3	23.1	21.7	24.5	22.9	21.9	23.9	26.0	24.4	27.7
2005	22.3	20.9	23.6	22.3	20.9	23.6	-	-	-	-	-	-
2006	15.3	14.9	15.7	19.5	18.2	21.0	13.9	13.4	14.3	21.9	20.4	23.5
2007	18.1	16.8	19.5	18.1	16.8	19.5	-	-	-	-	-	-
2008	13.7	13.2	14.3	17.1	15.7	18.5	12.7	12.1	13.4	-	-	-
2009	15.1	14.2	15.9	16.9	15.5	18.3	-	-	-	13.5	12.3	14.%
2010	11.3	11.0	11.6	15.2	13.9	16.6	11.0	10.7	11.3	-	-	-
2011	13.6	12.3	15.0	13.6	12.3	15.0	-	-	-	-	-	-
2012	14.3	13.1	15.6	14.3	13.1	15.6	-	-	-	-	-	-
2013	9.8	9.5	10.1	12.9	11.5	14.4	9.5	9.2	9.8	10.4	94.2	11.5
2014	11.5	10.3	12.9	11.5	10.3	12.9	-	-	-	-	-	-
2015	8.7	8.4	9.0	-	-	-	8.9	8.5	9.%	8.4	7.8	8.9

Table 12: Prevalence and CI (95%) of ever cannabis use between 1998-2015, by gender and school year, among students in England, Scotland and Wales

		95% CI			95% CI			95% CI			95% CI	
Year	**Males** (%)	Low	High	**Females** (%)	Low	High	**13 year olds** (%)	Low	High	**15 year olds** (%)	Low	High
1998	30.4	28.6	32.3	26.5	24.8	28.3	8.8	7.2	10.5	34.6	33.0	36.1
1999	23.5	21.6	25.4	19.2	17.3	21.1	9.8	8.5	11.2	34.0	31.7	36.2
2000	21.4	20.1	22.7	20.5	19.2	21.8	10.4	9.4	11.4	32.4	30.9	34.0
2001	27.1	25.1	29.2	23.0	21.0	25.0	14.7	13.2	16.4	37.2	34.8	39.5
2002	25.7	25.0	26.5	21.5	20.8	22.2	11.7	11.1	12.2	36.0	35.2	36.9
2003	28.0	26.0	30.1	23.7	21.8	25.6	13.2	11.8	14.8	39.8	37.6	42.1
2004	25.2	24.1	26.3	22.0	21.0	23.0	12.5	11.7	13.3	35.6	34.4	36.8
2005	23.2	21.3	25.2	21.3	19.4	23.3	10.9	9.5	12.3	35.9	33.5	38.2
2006	16.3	15.7	16.9	14.2	13.6	14.8	6.6	6.2	7.0	24.6	23.8	25.3
2007	18.5	16.6	20.6	17.7	15.8	19.7	7.5	6.3	8.9	29.2	26.9	31.6
2008	15.2	14.3	16.1	12.3	11.5	13.1	5.5	5.0	6.1	23.1	22.0	24.1
2009	15.7	14.4	17.0	14.4	13.2	15.7	6.3	5.5	7.1	24.9	23.4	26.5
2010	13.1	12.6	13.5	9.4	9.0	9.8	3.7	3.5	4.0	19.5	18.9	20.1
2011	15.9	13.9	18.1	11.6	9.9	13.4	6.2	4.9	7.6	21.6	19.3	24.0
2012	16.0	14.2	17.9	12.7	11.1	14.5	5.5	4.4	6.7	24.0	21.8	26.3
2013	10.8	10.3	11.2	8.7	8.3	9.1	3.3	3.0	3.5	16.7	16.1	17.2
2014	13.0	11.2	15.0	10.0	8.4	11.8	4.0	3.0	5.3	19.6	17.4	22.0
2015	10.1	9.6	10.5	7.4	7.0	7.8	3.1	2.9	3.4	15.3	14.7	15.9

Table 13: Prevalence and CI (95%) of ever alcohol use between 1998-2015, by year and by country, among students in England, Scotland and Wales

		95% CI			95% CI			95% CI			95% CI	
Year	**All** (%)	Low	High	**England** (%)	Low	High	**Scotland** (%)	Low	High	**Wales** (%)	Low	High
1998	78.9	77.5	80.2	81.3	79.4	83.0	76.2	74.1	78.2	-	-	-
1999	77.3	75.9	78.6	77.3	75.9	78.6	-	-	-	-	-	-
2000	73.5	72.2	74.7	75.3	73.7	76.9	71.3	69.4	73.1	-	-	-
2001	77.9	76.5	79.3	77.9	76.5	79.3	-	-	-	-	-	-
2002	79.5	79.1	80.0	77.3	75.9	78.6	78.5	77.9	79.0	93.2	92.1	94.2
2003	77.3	75.9	78.5	77.3	75.9	78.5	-	-	-	-	-	-
2004	78.7	78.0	79.4	75.5	74.1	76.9	77.7	76.7	78.7	85.3	83.9	86.6
2005	73.8	72.3	75.2	73.8	72.3	75.2	-	-	-	-	-	-
2006	72.2	71.7	72.7	70.7	69.1	72.3	71.2	70.6	71.8	81.9	80.4	83.3
2007	69.8	68.1	71.4	69.8	68.1	71.4	-	-	-	-	-	-
2008	66.9	66.1	67.7	69.3	67.6	70.9	66.2	65.3	67.1	-	-	-
2009	70.6	69.4	71.7	67.8	66.1	69.5	-	-	-	72.9	71.4	74.3
2010	60.1	59.6	60.5	61.5	59.7	63.3	60.0	59.5	60.5	-	-	-
2011	61.0	59.0	62.9	61.0	59.0	62.9	-	-	-	-	-	-
2012	58.6	56.9	60.4	58.6	56.9	60.4	-	-	-	-	-	-
2013	50.5	50.0	51.0	54.1	52.0	56.3	49.5	49.0	50.1	57.8	56.1	59.5
2014	51.7	49.7	53.8	51.7	49.7	53.8	-	-	-	-	-	-
2015	47.7	47.1	48.2	-	-	-	44.7	44.0	45.3	54.3	53.3	55.2

Table 14: Prevalence and CI (95%) of ever alcohol use between 1998-2015, by gender and year group, among students in England, Scotland and Wales

		95% CI			95% CI			95% CI			95% CI	
Year	**Males** (%)	Low	High	**Females** (%)	Low	High	**13 year olds** (%)	Low	High	**15 year olds** (%)	Low	High
1998	79.8	77.9	81.6	78.0	76.0	79.8	61.1	58.3	64.0	87.1	85.7	88.4
1999	77.9	76.0	79.7	76.6	74.5	78.6	66.3	64.1	68.4	89.1	87.6	90.6
2000	73.5	71.8	75.2	73.4	71.6	75.1	61.4	59.5	63.2	86.3	84.9	87.6
2001	77.4	75.4	79.3	78.4	76.4	80.3	69.0	66.9	71.1	88.3	86.6	89.8
2002	78.8	78.1	79.5	80.3	79.6	80.9	71.0	70.3	71.7	89.4	88.9	89.9
2003	76.7	74.8	78.5	77.8	75.9	79.7	67.5	65.4	69.5	88.3	86.7	89.7
2004	76.8	75.7	77.8	80.5	79.6	81.5	69.7	68.6	70.8	88.5	87.6	89.2
2005	72.0	69.9	74.1	75.7	73.6	77.7	62.5	60.3	64.6	87.3	85.6	88.9
2006	71.2	70.4	71.9	73.2	72.5	73.9	59.7	58.9	60.5	85.5	84.9	86.1
2007	68.9	66.6	71.2	70.6	68.2	72.9	57.1	54.6	59.6	83.0	81.0	84.9
2008	66.6	65.4	67.8	67.2	66.0	68.3	53.4	52.3	54.6	82.2	81.2	83.1
2009	70.9	69.4	72.5	70.2	68.5	71.7	58.5	56.8	60.1	84.1	82.8	85.4
2010	59.8	59.1	60.5	60.3	59.7	61.0	44.1	43.4	44.8	77.4	76.8	78.0
2011	61.4	58.6	64.2	60.6	57.9	63.2	45.8	43.1	48.6	77.1	74.6	79.4
2012	58.8	56.3	61.3	58.5	55.9	61.0	42.1	39.6	44.6	76.7	74.5	78.9
2013	49.9	49.2	50.6	51.1	50.3	51.8	32.4	31.8	33.0	69.9	69.3	70.6
2014	50.4	47.6	53.3	53.1	50.2	56.0	32.4	29.8	35.1	72.5	69.9	75.1
2015	47.2	46.5	48.0	47.9	47.2	48.7	31.1	30.4	31.7	66.9	66.2	67.6

Additional subgroup analyses for models

Table 15: Odd ratios of linear and quadratic models for ever smoked and regular smoking between 1998-2015, by country and socioeconomic status, among students in England, Scotland and Wales

		Ever smoked		Regular smoking	
		Linear	Quadratic	Linear	Quadratic
England (n = 51,923; 51,923)	Year	**0.93 [0.93-0.94]**	**1.03 [1.01- 1.06]**	**0.94 [0.93-0.95]**	**1.04 [1.01-1.08]**
	$Year^2$	-	**0.99 [0.99-0.99]**	-	**0.99 [0.99-1.00]**
	Level	0.90 [0.81-1.01]	1.02 [0.91-1.14]	**0.73 [0.59-0.89]**	0.85 [0.69-1.05]
	Post-slope	**0.93 [0.89-0.97]**	1.04 [0.99-1.10]	0.98 [0.90-1.05]	**1.11 [1.02-1.21]**
Receive FSM [a] (n = 17,574; 17,574)	Year	**0.94 [0.93-0.96]**	1.02 [0.97-1.08]	**0.95 [0.94-0.97]**	1.01 [0.94-1.09]
	$Year^2$	-	**1.00 [0.99-1.00]**	-	1.00 [0.99-1.00]
	Level	0.87 [0.66-1.15]	0.90 [0.68-1.19]	0.78 [0.50-1.21]	0.80 [0.52-1.25]
	Post-slope	0.94 [0.86-1.03]	1.01 [0.91-1.12]	0.98 [0.84-1.14]	1.03 [0.88-1.21]
Do not receive FSM [a] (n = 130,138; 130,138)	Year	**0.92 [0.92-0.93]**	**0.97 [0.95-0.99]**	**0.93 [0.93-0.94]**	0.97 [0.94-1.00]
	$Year^2$	-	**1.00 [1.00-1.00]**	-	**1.00 [1.00-1.00]**
	Level	0.95 [0.84-1.08]	0.97 [0.85-1.09]	**0.73 [0.58-0.92]**	**0.74 [0.59-0.94]**
	Post-slope	**0.92 [0.88-0.95]**	**0.95 [0.91-1.00]**	0.99 [0.92-1.07]	1.03 [0.94-1.11]
High FAS [b] (n = 10,299; 10,345)	Year	**0.86 [0.84-0.88]**	1.09 [0.89-1.33]	**0.90 [0.87-0.93]**	1.26 [0.93-1.71]
	$Year^2$	-	**0.99 [0.97-1.00]**	-	**0.98 [0.96-1.00]**
	Level	1.33 [0.92-1.93]	1.30 [0.90-1.89]	**0.46 [0.22-0.96]**	**0.46 [0.22-0.95]**
	Post-slope	0.96 [0.88-1.04]	1.22 [0.98-1.52]	1.14 [0.98-1.34]	**1.63 [1.15-2.33]**
Low FAS [b] (n = 11,427; 11,516)	Year	**0.88 [0.86-0.90]**	1.18 [0.97-1.43]	**0.90 [0.87-0.93]**	1.21 [0.93-1.58]
	$Year^2$	-	**0.98 [0.97-0.99]**	-	**0.98 [0.97-1.00]**
	Level	1.13 [0.81-1.56]	1.07 [0.77-1.49]	0.96 [0.57-1.62]	0.93 [0.55-1.57]
	Post-slope	0.99 [0.92-1.06]	**1.33 [1.08-1.64]**	1.03 [0.92-1.16]	**1.40 [1.04-1.89]**
SIMD least deprived[c] (n = 53,319; 53,319)	Year	**0.97 [0.95-0.98]**	**1.62 [1.07-2.46]**	1.02 [0.99-1.05]	**3.53 [1.69-7.38]**
	$Year^2$	-	**0.98 [0.96-1.00]**	-	**0.95 [0.91-0.98]**
	Level	**0.65 [0.57-0.75]**	**0.47 [0.34-0.63]**	0.79 [0.61-1.03]	**0.35 [0.20-0.61]**
	Post-slope	1.00 [0.97-1.04]	**1.32 [1.05-1.66]**	**0.91 [0.84-0.98]**	**1.77 [1.19-2.64]**
SIMD most deprived[c] (n = 66,506; 66,506)	Year	0.93 [0.92-0.95]	**1.94 [1.38-2.72]**	**0.97 [0.95-0.99]**	**2.06 [1.20-3.54]**
	$Year^2$	-	**0.97 [0.95-0.98]**	-	**0.97 [0.94-0.99]**
	Level	0.79 [0.70-0.88]	**0.49 [0.38-0.62]**	0.89 [0.72-1.10]	**0.54 [0.36-0.82]**
	Post-slope	0.97 [0.94-1.00]	**1.43 [1.19-1.72]**	**0.90 [0.85-0.96]**	**1.35 [1.00-1.81]**

[a]Scotland and England data only [b] Wales data only [c] Scotland data only

Table 16: Odd ratios of linear and quadratic models for young people who think trying smoking is 'OK' (between 2001-2015), as well as smoking weekly is 'OK' (between 2003-2014), by country and socioeconomic status, among students in England and Scotland

		Trying smoking is "OK" [a] (don't know = not OK)		Smoking weekly OK [b] (don't know = not OK)	
		Linear	Quadratic	Linear	Quadratic
England (n = 43,117; 35,890)					
	Year	**0.90 [0.90-0.91]**	**0.85 [0.82-0.88]**	**0.91 [0.90-0.92]**	**0.72 [0.65-0.81]**
	$Year^2$	-	**1.00 [1.00-1.01]**	**-**	**1.01 [1.01-1.02]**
	Level	**1.25 [1.12-1.39]**	**1.18 [1.06-1.32]**	**1.15 [1.00-1.32]**	1.10 [0.96-1.27]
	Post-slope	**0.94 [0.90-0.97]**	**0.88 [0.83-0.92]**	**0.95 [0.90-1.00]**	**0.82 [0.75-0.89]**
Receive FSM[a] (n = 16,229; 4,889)					
	Year	**0.93 [0.92-0.95]**	**0.87 [0.81-0.93]**	**0.93 [0.90-0.96]**	0.93 [0.69-1.27]
	$Year^2$	-	**1.00 [1.00-1.01]**		1.00 [0.98-1.02]
	Level	1.10 [0.83-1.47]	1.09 [0.82-1.45]	1.20 [0.86-1.67]	1.20 [0.85-1.68]
	Post-slope	**0.90 [0.82-0.99]**	**0.85 [0.77-0.95]**	0.96 [0.85-1.08]	0.96 [0.76-1.21]
Do not receive FSM[a] (n = 122,533; 30,617)					
	Year	**0.91 [0.90-0.91]**	**0.86 [0.84-0.89]**	**0.90 [0.89-0.92]**	**0.70 [0.62-0.79]**
	$Year^2$	-	**1.00 [1.00-1.01]**	-	**1.01 [1.01-1.02]**
	Level	**1.18 [1.05-1.33]**	**1.18 [1.05-1.33]**	1.14 [0.98-1.33]	1.09 [0.94-1.27]
	Post-slope	**0.87 [0.84-0.91]**	**0.84 [0.81-0.88]**	**0.94 [0.89-1.00]**	**0.80 [0.72-0.88]**
SIMD least deprived[c] (n = 51,948)					
	Year	**0.92 [0.90-0.93]**	0.79 [0.53-1.17]	-	-
	$Year^2$	-	1.01 [0.99-1.03]	-	-
	Level	**0.76 [0.68-0.86]**	0.84 [0.63-1.12]	-	-
	Post-slope	0.97 [0.94-1.00]	0.89 [0.72-1.11]	-	-
SIMD most deprived[c] (n = 63,628)					
	Year	**0.92 [0.91-0.93]**	**0.64 [0.45-0.90]**	-	-
	$Year^2$	-	**1.02 [1.00-1.03]**	-	-
	Level	0.97 [0.87-1.08]	1.23 [0.96-1.59]	-	-
	Post-slope	**0.92 [0.89-0.95]**	**0.76 [0.63-0.91]**	-	-

[a] Scotland and England data only [b] England data only [c] Scotland data only

Table 17: Odd ratios of linear and quadratic models for young people who think trying smoking is "OK" (don't know = OK), as well as by gender, year group and socioeconomic status, among students in England and Scotland between 2001-2015

		Trying smoking is "OK" [a] (don't know = OK)	
		Linear	Quadratic
Whole Sample (n = 165,199)	Year	**0.92 [0.91-0.92]**	**0.84 [0.82- 0.86]**
	$Year^2$	-	**1.00 [1.00-1.01]**
	Level	**0.92 [0.87-0.98]**	0.95 [0.89-1.01]
	Post-slope	**0.98 [0.97-1.00]**	**0.91 [0.89-0.93]**
Males (n = 82,270)	Year	**0.93 [0.92-0.93]**	**0.81 [0.79-0.84]**
	$Year^2$	-	**1.01 [1.01-1.01]**
	Level	0.94 [0.86-1.02]	0.98 [0.90-1.07]
	Post-slope	**0.97 [0.95-0.99]**	**0.87 [0.84-0.90]**
Females (n = 82,929)	Year	**0.90 [0.90-0.91]**	**0.86 [0.83-0.89]**
	$Year^2$	-	**1.00 [1.00-1.00]**
	Level	**0.90 [0.83-0.98]**	**0.92 [0.84-1.00]**
	Post-slope	0.99 [0.97-1.01]	**0.95 [0.92-0.99]**
13 year olds (n = 85,713)	Year	**0.91 [0.90-0.92]**	**0.85 [0.82-0.88]**
	$Year^2$	-	**1.00 [1.00-1.01]**
	Level	**0.85 [0.78-0.93]**	**0.87 [0.80-0.95]**
	Post-slope	1.01 [0.99-1.03]	**0.96 [0.92-0.99]**
15 year olds (n = 79,486)	Year	**0.93 [0.92-0.94]**	**0.83 [0.80-0.86]**
	$Year^2$	-	**1.01 [1.00-1.01]**
	Level	0.99 [0.91-1.08]	1.04 [0.95-1.13]
	Post-slope	**0.94 [0.92-0.96]**	**0.86 [0.82-0.89]**
England (n = 43,177)	Year	**0.90 [0.90-0.91]**	**0.82 [0.79-0.85]**
	$Year^2$	-	**1.01 [1.00-1.01]**
	Level	**1.28 [1.15-1.43]**	**1.18 [1.06-1.32]**
	Post-slope	**0.93 [0.90-0.97]**	**0.84 [0.80-0.89]**
Receive FSM [a] (n = 16,229)	Year	**0.94 [0.93-0.96]**	**0.87 [0.82-0.94]**
	$Year^2$	-	**1.00 [1.00-1.01]**
	Level	1.02 [0.77-1.34]	1.01 [0.77-1.33]
	Post-slope	0.94 [0.85-1.03]	**0.88 [0.80-0.98]**
Do not receive FSM[a] (n = 122,533)	Year	**0.91 [0.90-0.92]**	**0.83 [0.81-0.86]**
	$Year^2$	-	**1.00 [1.00-1.01]**
	Level	**1.23 [1.10-1.38]**	**1.24 [1.10-1.39]**
	Post-slope	**0.89 [0.85-0.92]**	**0.83 [0.80-0.87]**
SIMD least deprived[b] (n = 51,948)	Year	**0.93 [0.91-0.94]**	1.17 [0.79-1.75]
	$Year^2$	-	0.99 [0.97-1.01]
	Level	**0.68 [0.61-0.76]**	**0.58 [0.44-0.77]**
	Post-slope	**1.03 [1.00-1.06]**	1.17 [0.94-1.45]
SIMD most deprived[b] (n = 63,628)	Year	**0.94 [0.93-0.96]**	0.79 [0.56-1.11]
	$Year^2$	-	1.01 [0.99-1.02]
	Level	**0.85 [0.77-0.94]**	0.96 [0.75-1.23]
	Post-slope	**0.96 [0.94-0.99]**	0.87 [0.73-1.05]

[a] Scotland and England data only [b]Scotland data only

Table 18: Odd ratios of linear and quadratic models for students in England who think smoking weekly is "OK" (don't know = OK), as well as by gender, year group and socioeconomic status

		Smoking weekly is "OK" (don't know = OK)	
		Linear	Quadratic
Whole Sample (n = 35,890)	Year	**0.92 [0.91-0.94]**	**0.72 [0.64-0.80]**
	$Year^2$	-	**1.01 [1.01-1.02]**
	Level	1.11 [0.99-1.25]	1.07 [0.95-1.21]
	Post-slope	**0.94 [0.90-0.98]**	**0.80 [0.74-0.86]**
Males (n =18,042)	Year	**0.94 [0.93-0.96]**	**0.69 [0.59-0.80]**
	$Year^2$	**-**	**1.02 [1.01-1.03]**
	Level	**1.26 [1.06-1.50]**	**1.20 [1.01-1.43]**
	Post-slope	**0.87 [0.81-0.92]**	**0.70 [0.63-0.79]**
Females (n = 17,848)	Year	**0.91 [0.89-0.92]**	**0.74 [0.64-0.86]**
	$Year^2$	**-**	**1.01 [1.00-1.02]**
	Level	1.00 [0.85-1.18]	0.97 [0.83-1.15]
	Post-slope	1.01 [0.96-1.08]	**0.89 [0.80-1.00]**
13 year olds (n = 18,721)	Year	**0.93 [0.92-0.95]**	**0.72 [0.62-0.84]**
	$Year^2$	**-**	**1.01 [1.01-1.02]**
	Level	1.14 [0.95-1.36]	1.09 [0.91-1.30]
	Post-slope	**0.93 [0.87-0.99]**	**0.78 [0.70-0.88]**
15 year olds (n = 17,169)	Year	**0.92 [0.90-0.93]**	**0.71 [0.61-0.82]**
	$Year^2$	**-**	**1.01 [1.01-1.02]**
	Level	1.10 [0.94-1.29]	1.06 [0.91-1.24]
	Post-slope	0.95 [0.90-1.01]	**0.81 [0.72-0.90]**
Receive FSM (n = 4,889)	Year	**0.94 [0.91-0.97]**	0.83 [0.99-1.02]
	$Year^2$	-	1.01 [0.99-1.02]
	Level	1.12 [0.83-1.51]	1.10 [0.81-1.49]
	Post-slope	0.94 [0.85-1.05]	0.87 [0.71-1.08]
Do not receive FSM (n = 30,617)	Year	**0.92 [0.91-0.93]**	**0.70 [0.62-0.79]**
	$Year^2$	-	**1.01 [1.01-1.02]**
	Level	1.12 [0.98-1.27]	1.07 [0.94-1.22]
	Post-slope	**0.94 [0.90-0.98]**	**0.79 [0.72-0.86]**

Table 19: Odd ratios of linear and quadratic models for ever drunk alcohol and ever cannabis use between 1998-2015 by country and socioeconomic status, among students in England, Scotland and Wales

		Ever drunk alcohol		Ever used cannabis	
		Linear	Quadratic	Linear	Quadratic
England (n = 51,949; 51,811)	Year	**0.94 [0.93-0.94]**	**1.07 [1.04-1.10]**	**0.96 [0.96-0.97]**	**1.15 [1.12-1.19]**
	Year^2	-	**0.99 [0.99-0.99]**	-	**0.99 [0.98-0.99]**
	Level	0.91 [0.81-1.01]	1.04 [0.93-1.16]	**0.82 [0.71-0.95]**	1.06 [0.91-1.24]
	Post-slope	**0.92 [0.89-0.96]**	**1.07 [1.01-1.12]**	0.96 [0.91-1.02]	**1.20 [1.13-1.28]**
Receive FSM [a] (n =17,724; 17,610)	Year	**0.95 [0.94-0.96]**	1.05 [0.99-1.11]	**0.95 [0.94-0.97]**	**1.08 [1.01-1.15]**
	Year^2	-	**0.99 [0.99-1.00]**	-	**0.99 [0.99-1.00]**
	Level	0.81 [0.61-1.07]	0.83 [0.63-1.10]	1.04 [0.73-1.48]	1.10 [0.77-1.57]
	Post-slope	0.95 [0.86-1.04]	1.03 [0.93-1.14]	0.96 [0.85-1.08]	1.07 [0.93-1.22]
Do not receive FSM [a] (n=131,173; 130,645)	Year	**0.91 [0.91-0.92]**	**1.09 [1.06-1.12]**	**0.94 [0.93-0.94]**	**1.05 [1.03-1.08]**
	Year^2	-	**0.99 [0.99-0.99]**	-	**0.99 [0.99-0.99]**
	Level	1.01 [0.90-1.14]	1.03 [0.92-1.16]	0.89 [0.75-1.04]	0.93 [0.79-1.10]
	Post-slope	**0.91 [0.88-0.95]**	**1.05 [1.00-1.09]**	1.01 [0.96-1.07]	**1.12 [1.06-1.19]**
High FAS [b] (n =10,238; 9,135)	Year	**0.79 [0.76-0.82]**	**0.57 [0.43-0.76]**	**0.90 [0.87-0.93]**	**1.71 [1.31-2.23]**
	Year^2	-	**1.02 [1.00-1.03]**	-	**0.96 [0.95-0.98]**
	Level	0.85 [0.62-1.18]	0.93 [0.67-1.30]	1.02 [0.62-1.68]	0.96 [0.58-1.59]
	Post-slope	**1.15 [1.07-1.24]**	0.86 [0.66-1.12]	0.97 [0.87-1.09]	**1.84 [1.39-2.45]**
Low FAS [b] (n = 11,393; 10,258)	Year	**0.80 [0.78-0.83]**	**0.66 [0.50-0.86]**	**0.92 [0.89-0.95]**	**1.49 [1.16-1.90]**
	Year^2	-	1.01 [1.00-1.03]	-	**0.97 [0.96-0.99]**
	Level	0.79 [0.59-1.05]	0.84 [0.62-1.13]	1.17 [0.77-1.79]	1.08 [0.70-1.65]
	Post-slope	**1.17 [1.09-1.25]**	0.96 [0.75-1.24]	0.94 [0.85-1.03]	**1.51 [1.16-1.96]**
SIMD least deprived[c] (n = 53,566; 53,522)	Year	**0.88 [0.86-0.89]**	**1.53 [1.01-2.30]**	0.99 [0.96-1.01]	**2.60 [1.41-4.80]**
	Year^2	-	**0.98 [0.96-0.99]**	-	**0.96 [0.93-0.98]**
	Level	**0.76 [0.68-0.85]**	**0.53 [0.39-0.70]**	**0.73 [0.60-0.88]**	**0.39 [0.25-0.60]**
	Post-slope	**1.06 [1.03-1.10]**	**1.43 [1.15-1.79]**	**1.05 [1.00-1.11]**	**1.77 [1.27-2.47]**
SIMD most deprived[c] (n = 66,912; 66,850)	Year	**0.87 [0.86-0.89]**	1.14 [0.79-1.63]	**0.96 [0.95-0.98]**	**2.41 [1.49-3.90]**
	Year^2	-	0.99 [0.97-1.00]	-	**0.96 [0.94-0.98]**
	Level	**0.89 [0.80-0.98]**	**0.75 [0.58-0.96]**	1.01 [0.86-1.19]	**0.55 [0.39-0.79]**
	Post-slope	1.01 [0.98-1.04]	1.16 [0.95-1.41]	0.97 [0.93-1.01]	**1.59 [1.22-2.06]**

[a] Scotland and England only; [b] Wales only [c] Scotland only

Table 20: Odd ratios of quadratic models for interaction terms (gender and year group), for all outcomes among students in England, Scotland and Wales

Interaction terms	Ever smoked (n = 242,855)	Weekly smoking (n = 243,111)	Smoking OK[a] [Don't know = not OK] (n = 165,199)	Smoking weekly OK[b] [Don't know = not OK] (n = 35,890)	Ever alcohol use (n = 239,190)	Ever cannabis use (n = 239,457)
Year*gender	0**.97 [0.97-0.98]**	**0.98 [0.97-0.99]**	**0.98 [0.97-0.99]**	**0.97 [0.94-0.99]**	0.99 [0.99-1.00]	**0.99 [0.98-0.99]**
Level*gender	0.99 [0.88-1.12]	0.88 [0.70-1.10]	0.96 [0.86-1.09]	**0.76 [0.58-1.00]**	1.02 [0.92-1.14]	1.14 [0.96-1.35]
Trend*gender	1.01 [0.98-1.04]	1.00 [0.94-1.05]	1.01 [0.98-1.04]	**1.15 [1.05-1.27]**	1.00 [0.97-1.02]	0.98 [0.94-1.02]
Year*year group	**1.04 [1.03-1.05]**	**1.04 [1.03-1.05]**	**1.04 [1.03-1.05]**	1.00 [0.98-1.03]	**1.02 [1.01-1.03]**	**1.04 [1.03-1.05]**
Level*year group	**1.17 [1.03-1.32]**	1.21 [0.93-1.58]	1.08 [0.95-1.21]	0.91 [0.69-1.21]	**1.12 [1.01-1.25]**	0.94 [0.77-1.15]
Trend*year group	**0.96 [0.93-0.99]**	**0.93 [0.87-0.99]**	**0.95 [0.92-0.98]**	1.03 [0.93-1.14]	0.98 [0.95-1.00]	0.99 [0.94-1.04]

[a] England and Scotland data only [b] England data only

Figure 1: Predicted probabilities of ‘ever smoking’ from 1998-2015 by gender and year group, in England, Scotland and Wales from logistic regression analyses

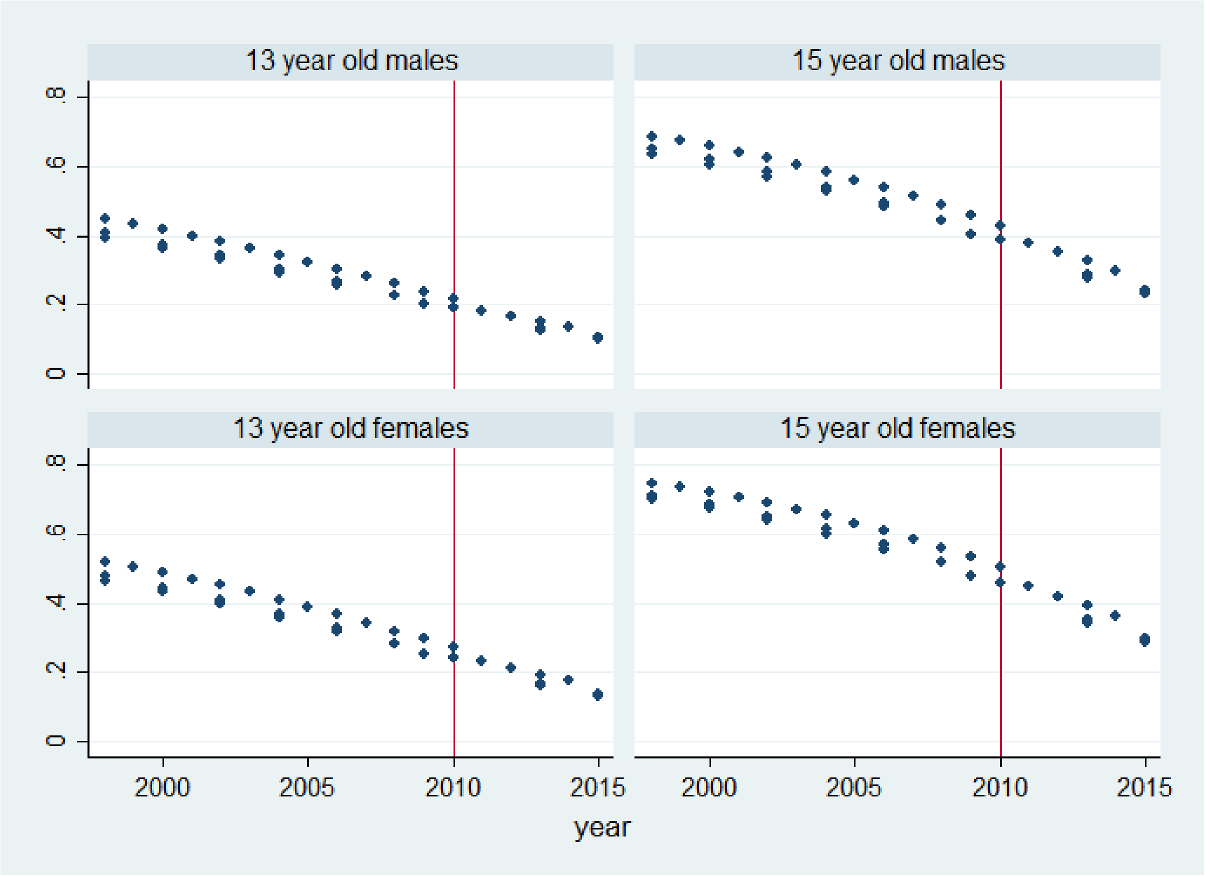

Figure 2: Predicted probabilities of regular smoking from 1998-2015 by gender and year group, in England, Scotland and Wales from logistic regression analyses

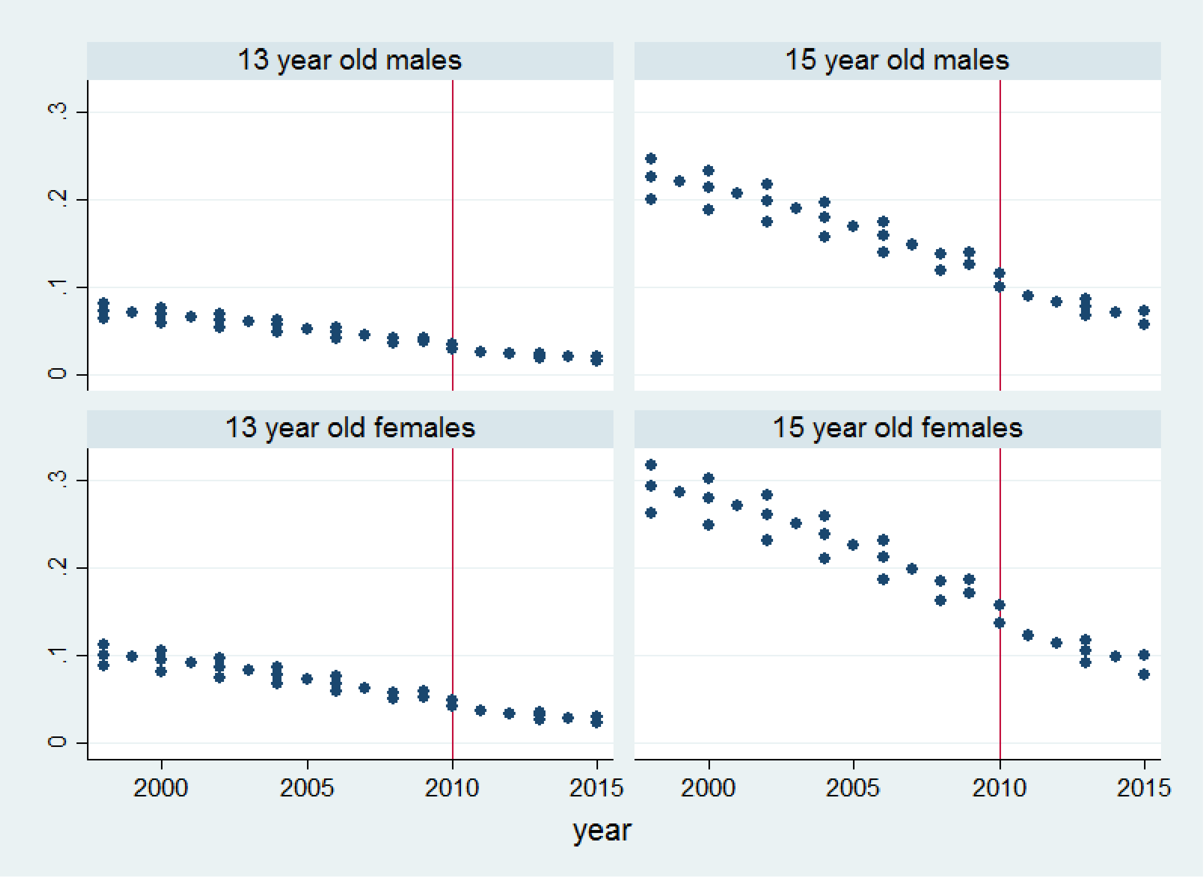

Figure 3: Predicted probabilities of stating that trying smoking is "OK" (don't know = no) from 2001-2015 by gender and year group, in England and Scotland, from binary logistic regression analyses

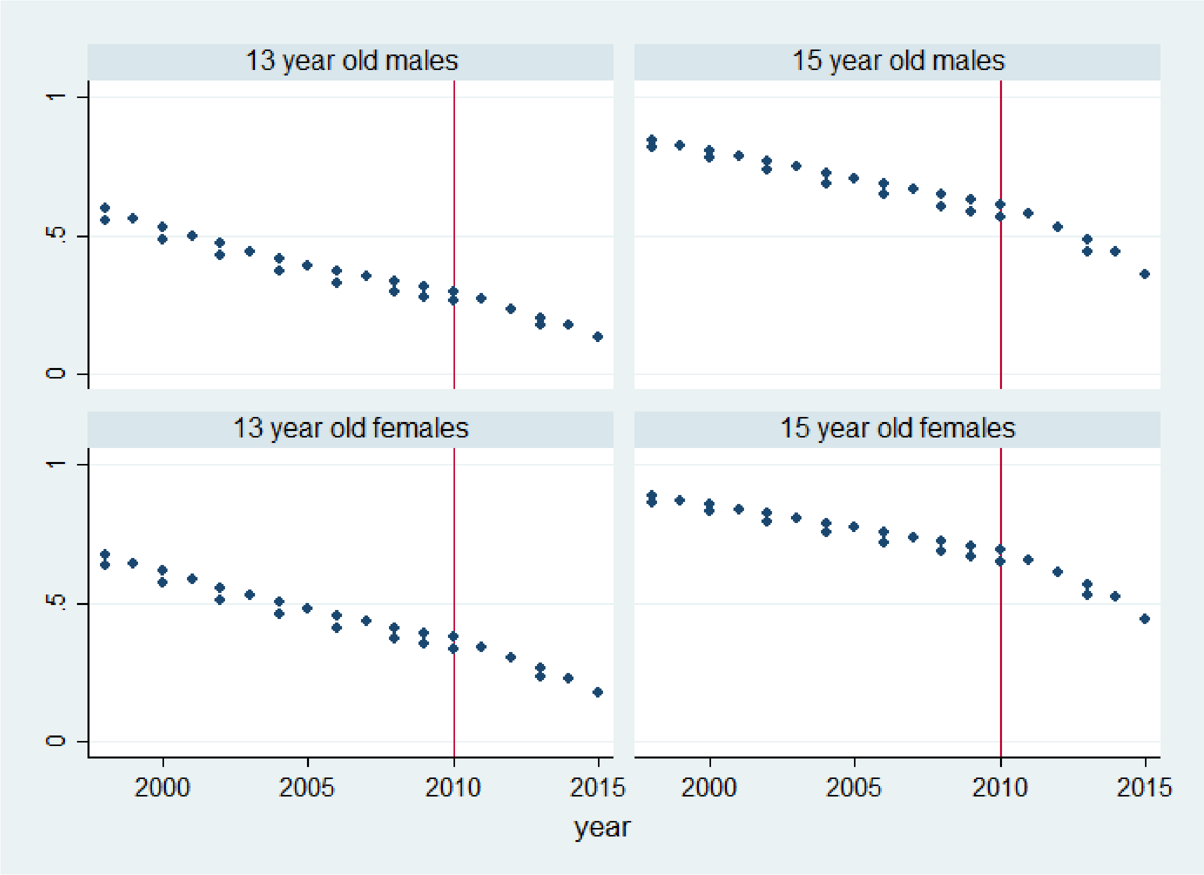

Figure 4: Predicted probabilities of stating that smoking weekly is “OK” (don’t know = no) from 2003-2014 by gender and year group, in England, from binary logistic regression analyses

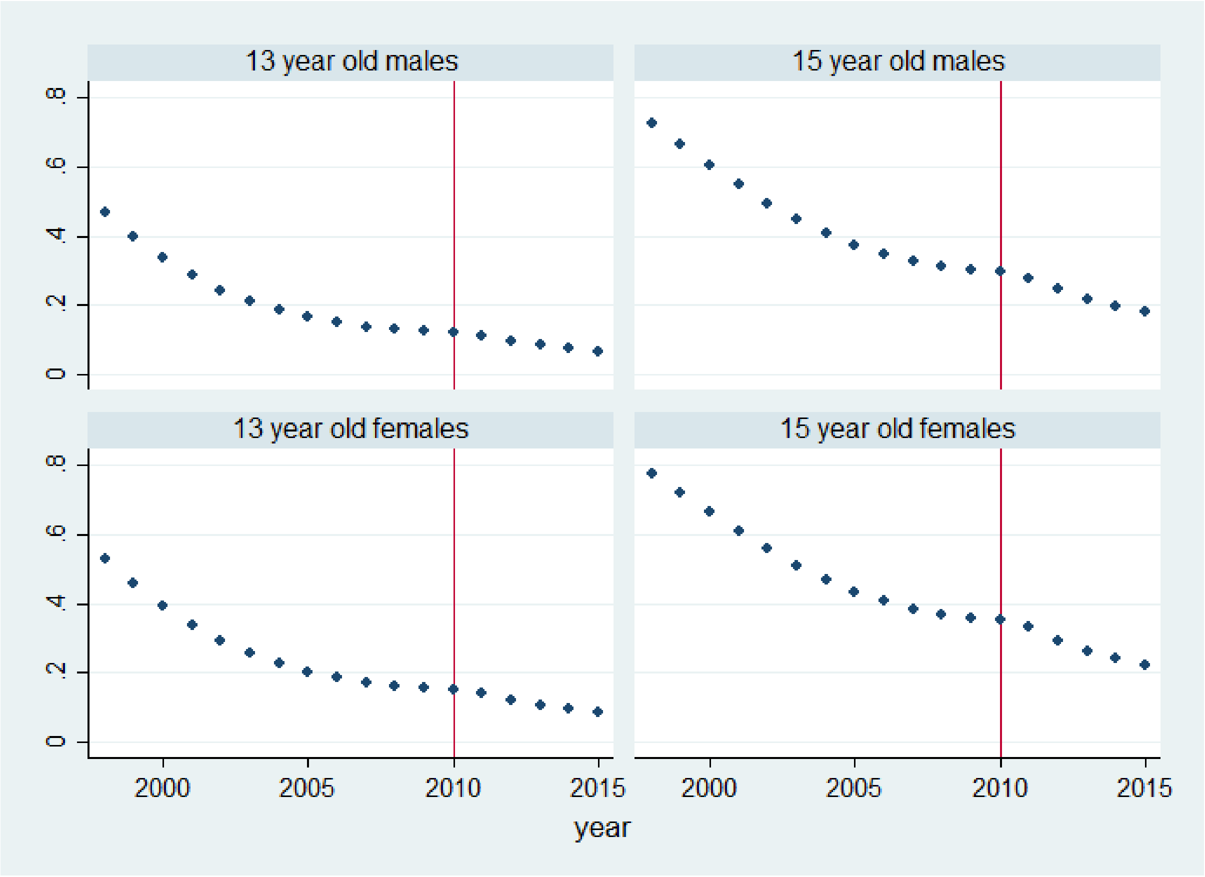

Figure 5: Predicted probabilities of ever alcohol use from 1998-2015 by gender and year group, in England, Scotland and Wales, from binary logistic regression analyses

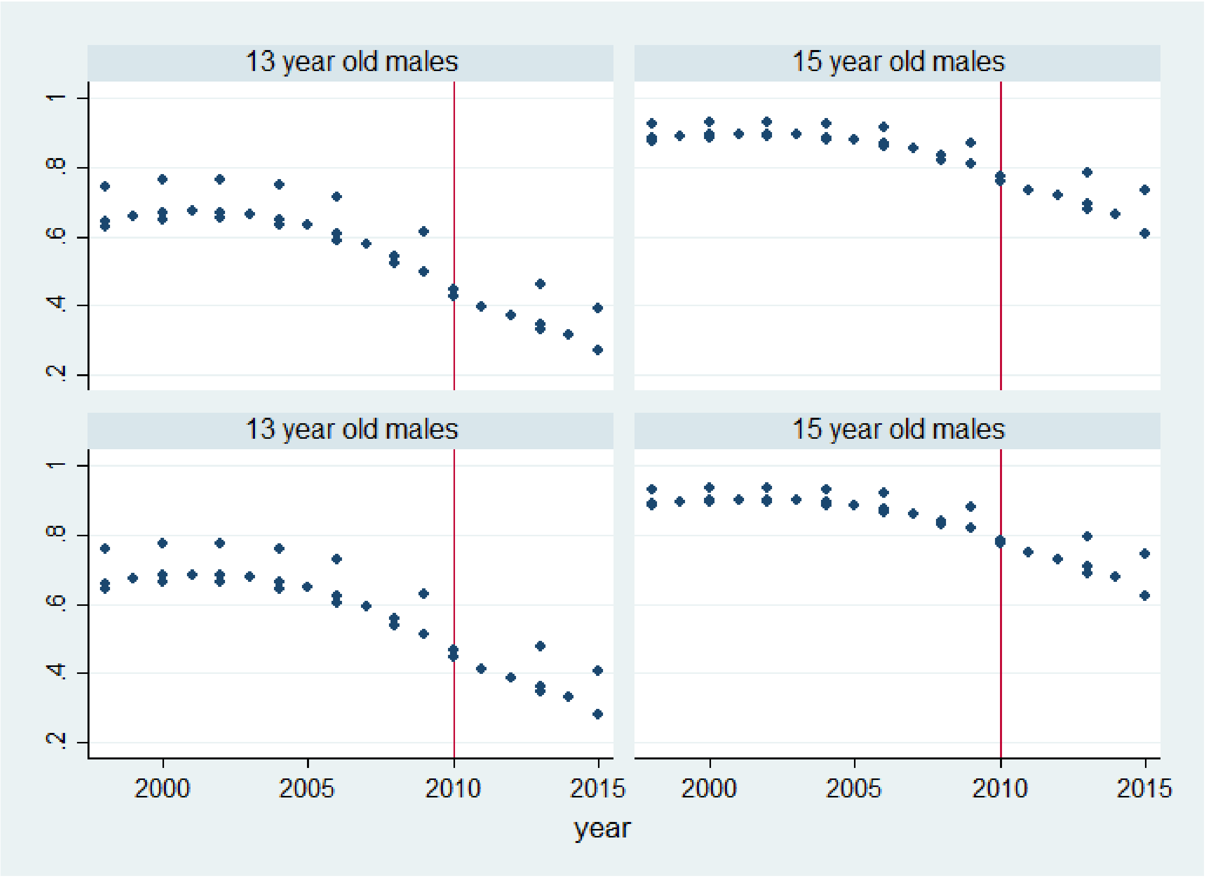

Figure 6: Predicted probabilities of ever cannabis use from 1998-2015 by gender and year group, in England, Scotland and Wales, from binary logistic regression analyses

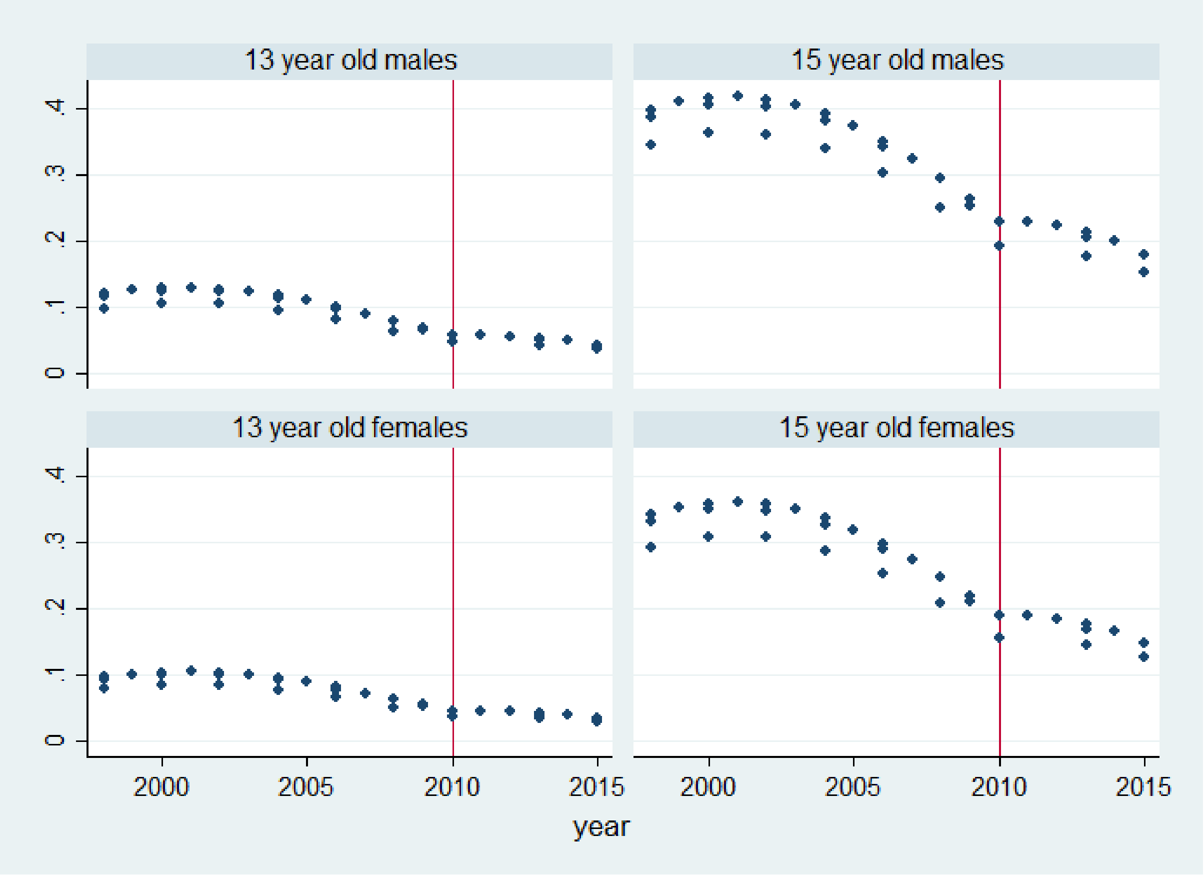

Supplement: Supplementary data [file tobaccocontrol-2018-054584supp001.pdf]
